# A Complex Cell Division Machinery Was Present in the Last Common Ancestor of Eukaryotes

**DOI:** 10.1371/journal.pone.0005021

**Published:** 2009-04-07

**Authors:** Laura Eme, David Moreira, Emmanuel Talla, Céline Brochier-Armanet

**Affiliations:** 1 Laboratoire de Chimie Bacterienne – UPR CNRS 9043, Marseille, France; 2 Unité d'Ecologie, Systématique et Evolution – UMR CNRS 8079, Université Paris-Sud, Orsay, France; 3 Université de la Méditerranée, Marseille, France; 4 Université de Provence, Marseille, France; University of California, Berkeley, United States of America

## Abstract

**Background:**

The midbody is a transient complex structure containing proteins involved in cytokinesis. Up to now, it has been described only in Metazoa. Other eukaryotes present a variety of structures implied in the last steps of cell division, such as the septum in fungi or the phragmoplast in plants. However, it is unclear whether these structures are homologous (derive from a common ancestral structure) or analogous (have distinct evolutionary origins). Recently, the proteome of the hamster midbody has been characterized and 160 proteins identified.

**Methodology/Principal Findings:**

Using phylogenomic approaches, we show here that nearly all of these 160 proteins (95%) are conserved across metazoan lineages. More surprisingly, we show that a large part of the mammalian midbody components (91 proteins) were already present in the last common ancestor of all eukaryotes (LECA) and were most likely involved in the construction of a complex multi-protein assemblage acting in cell division.

**Conclusions/Significance:**

Our results indicate that the midbodies of non-mammalian metazoa are likely very similar to the mammalian one and that the ancestor of Metazoa possessed a nearly modern midbody. Moreover, our analyses support the hypothesis that the midbody and the structures involved in cytokinesis in other eukaryotes derive from a large and complex structure present in LECA, likely involved in cytokinesis. This is an additional argument in favour of the idea of a complex ancestor for all contemporary eukaryotes.

## Introduction

The presence of large multiprotein complexes has been often considered as one of the main distinctive differences between eukaryotic and prokaryotic cells. These Eukaryotic Multiprotein Complexes (EMC), such as the nuclear pore complex associated to nuclear membranes [1], the spliceosome [2], the telomeric complex [3], and the nucleolus [4], are involved in various cellular processes or structures. Surprisingly, even if similar cellular processes exist in prokaryotes, these appear to be fulfilled by simpler and often non homologous systems [5], [6]. This suggests that the appearance of EMC was a eukaryotic-specific evolutionary trend. Despite their importance to understand eukaryotic evolution, the evolutionary origin of many EMC remains elusive. In fact, most studies of EMC have focused on the functional characterization of some of their components rather than on evolutionary considerations.

Recently, thanks to the development of massive proteomic methods, a number of EMC, including the telomeric complex [7], the flagellum and the associated basal body [8], [9], or the centromere complex [10], have been characterized leading to the identification of most of their components. This, in parallel with the increasing availability of genomic data for diverse eukaryotic and prokaryotic species, has opened the possibility to carry out phylogenomic studies of these biological systems. This kind of analysis consists in identifying the homologues of each component involved in a biological system or cellular process in all lineages of the studied taxonomic group [11], [12]. The phylogenetic analysis of each component then allows determining its evolutionary origin as well as its subsequent evolutionary history (duplication events, losses, horizontal gene transfer (HGT)). The interpretation of functional data from experimental studies (e.g., mutant defects, interactome data, expression data) or from *in silico* analyses (e.g., functional domain and motif searches, genomic context analysis) in the light of the evolutionary framework obtained may help to infer functional predictions for organisms for which no such functional data are available, as well as to propose targets for experimental analyses. The recent availability of both proteomic and genomic data in eukaryotes has provided the necessary starting point for the first evolutionary studies of EMC [13], [14], [15]. This has allowed addressing questions such as when a particular EMC appeared and what was its ancestral composition, and to retrace its history (e.g., component acquisitions and losses) in the different lineages.

Among the different EMC known, those responsible of cytokinesis, the last step of the cell division, are particularly complex. First described in 1891 [16], the midbody is a transient organelle-like structure found in animal cells that forms a bridge between the two daughter cells at the end of the cytokinesis [17], [18], [19], [20]. It consists of a compact and dense matrix of proteins of 1–1,5 µm in diameter, tightly bundled by the cleavage furrow [21]. It is composed mainly of microtubules derived from the spindle midzone that are coated by microtubule-associated proteins that maintain the structure [17]. Golgi-derived vesicles, possibly involved in the transport of proteins and membranes, as well as proteins with secretory and membrane-traffic activities, have also been reported along and around the midbody microtubules [22]. A midbody association with endoplasmic reticulum structures has also been described in some animals, as *Drosophila melanogaster* and *Caenorhabditis elegans* (see [17] and references therein). At the end of the cytokinesis, the midbody is severed, leading to the separation of the daughter cells. Even if no clear function has been ascribed to the midbody, mutation or silencing of most of its components is associated to defects in cleavage furrow formation or completion and to defects in germline cytokinesis [22].

Up to now, the midbody has only been described in metazoans. Despite similarities at the mechanistic level, Metazoa, Fungi and other eukaryotes, present a variety of structures involved in the last steps of cell division. Consequently, it is unclear whether these structures are homologous (derived from a common ancestral structure) or analogous (have distinct evolutionary origins) [17], [23]. For example, the amoebozoan *Dictyostelium discoideum* produces cytoplasmic bridges that present some similarities with the animal midbody (see [20], [24], [25]), whereas Fungi exhibit a chitin-rich septum at the division site [26]. The most distinct cytokinesis structure analysed in detail, the phragmoplast, is found in land plants. It is a dense structure likely derived from the mitotic spindle by reassembling of microtubules. which become responsible of the targeted delivery of membrane vesicles for the formation of the division plate [19], [23], [27], [28]. Although it contains a certain amount of actin with an unclear role, the plant phragmoplast appears not to involve an actinomyosin contractile ring, in contrast with the animal midbody [28], and the division plate grows centrifugally. Both the midbody and the phragmoplast contain a number of microtubule-associated proteins and kinesin motor molecules that stabilize the structure [23]. Interestingly, in red algae, which are close relatives to the green plants (Figure S1), the presence of an actin contractile ring and furrowing have been reported [29], suggesting that the plant phragmoplast is a very derived structure. Accordingly, various situations have been described in green algae [27], [30]. In most green algae, cytokinesis involves furrowing from the cell cortex, but centrifugal division by means of a phragmoplast also exists (see [30] and [31] and references therein). Intermediate situations have also been described as in *Spirogyra*, in which cytokinesis is initiated by an actin-based cleavage furrow and completed by a phragmoplast-like array of microtubules. However, in contrast with land plants, that phragmoplast-like structure progresses towards the centre of the cell division plan and does not grow centrifugally [32].

A recent proteomic analysis has identified 160 different proteins in the midbody from Chinese hamster ovary cells [22]. As expected, most of them are involved in secretory and membrane traffic activities (53 proteins), are actin- or microtubule-associated proteins (46 and 18 proteins, respectively), and protein kinases (18 proteins). The remaining 25 proteins display a variety of functions [22] (Table S1). Using these proteins as a starting point, we have carried out a phylogenomic analysis of the mammalian midbody and we show that a midbody very similar in protein composition to the mammalian one was most likely present in the last common ancestor of Metazoa. We also show that a large fraction of the mammalian midbody components are ancient and were already present in the last eukaryotic common ancestor (LECA), even if they appear to have originated after the prokaryotes/eukaryotes separation. Moreover, taking into account available functional data and based on functional domains present in these proteins, it is possible to infer that in non-metazoan eukaryotes these proteins have similar molecular functions and are most likely also involved in the late step of cytokinesis. All this indicates that a complex cellular structure responsible of cell division was already present in LECA, from which likely all of the present-day eukaryotic cytokinesis structures derive.

## Results and Discussion

To start our analysis, we first retrieved from public databases all the homologues of each of the 160 proteins identified in the hamster midbody [22] (Table S1). To distinguish between orthologues and paralogues among all the homologues retrieved, we performed a phylogenetic analysis of each protein. This allowed an accurate identification of orthologues, which is an essential step of phylogenomic analyses because orthologues are more likely to share the same function than paralogues [11]. At this step, we discarded two proteins: CDK4 (kin6), because of the lack of phylogenetic signal to differentiate orthologues from paralogues, and Ankyrin B/Ank2 (act33), due to the presence of ankyrin repeats that distorted the multiple sequence alignment. Phylogenetic analyses of Dynamin 2, Dynamin-like I and Dynamin-like DLP1 (sec19, sec20 and sec21) and of Calnexin and Calreticulin (sec10 and sec12) showed that they resulted from two and one recent duplication events, respectively, which occurred in vertebrates and were thus gathered in two datasets (sec19/20/21 and sec10/12). 155 protein datasets were thus finally kept for further analyses. Our phylogenetic analyses showed a high number of duplications that are vertebrate-specific (more than one third of the midbody components had paralogues that were vertebrate-specific), in agreement with the proposal that the vertebrate lineage evolved through two successive rounds of complete genome duplication [33]. However, with the exception of the sec19/sec20/sec21 and sec10/sec12 cases mentioned above, the paralogues resulting from these duplication events were not part of the hamster midbody proteome. This suggests that functional divergences occurred after these duplication events.

### A modern-like midbody in the ancestor of Metazoa

**Figure 1:**
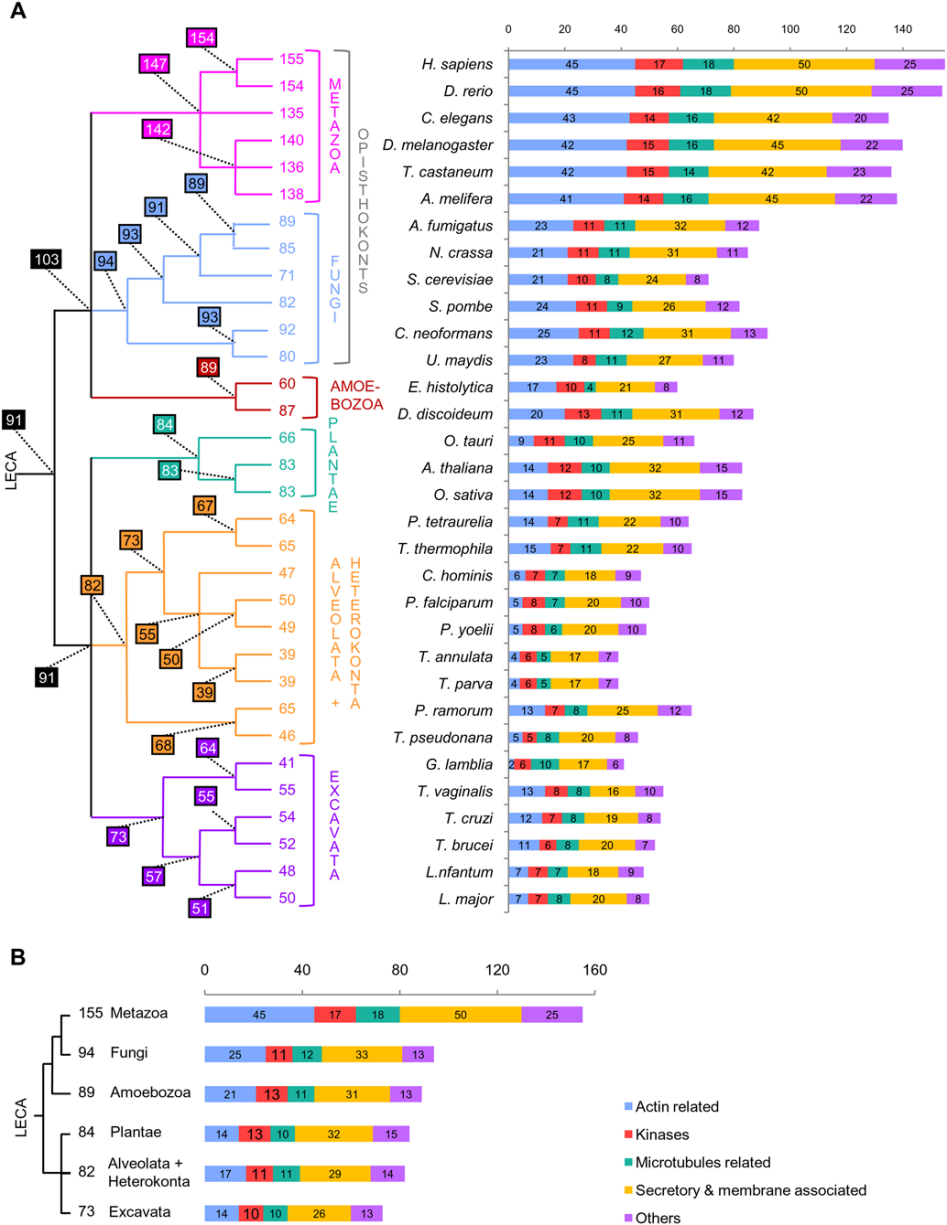
Distribution of the orthologues of the hamster midbody among eukaryotic lineages. Number of orthologues of the mammalian-midbody components present in each of the 32 eukaryotic species (A) and in the ancestor of each of the six main eukaryotic phyla (B). Colours represent the functional categories defined by Skop, et al. [22]. The phylogenetic relationships between the lineages analysed are indicated on the left. Numbers at nodes indicate the number of orthologues inferred to be present in the corresponding ancestors.

Based on the phylogenies of the 155 datasets retained, we identified as many as 140 and 135 orthologues in distant invertebrate species such as *Drosophila melanogaster* and *Caenorhabditis elegans*, respectively. This supported the hypothesis that the midbodies of these animals are very similar to the mammalian ones. In agreement with this result, a recent RNA interference-based inactivation analysis has showed that in *C. elegans*, ∼100 homologues of hamster midbody components are involved in cleavage furrow formation or completion, or in germ line cytokinesis [22]. This confirmed that the composition and function of the midbody are extremely well conserved in the whole animal phylum. We also inferred that 154 and 147 proteins were present in the ancestor of Vertebrata and in the ancestor of Metazoa, representing 99% and 95% of the hamster midbody components, respectively (Figure 1A). This implied that only eight components were recruited in the lineage leading from the ancestor of Metazoa to mammals and suggested that the ancestor of living Metazoa had already a midbody with modern characteristics.

### Most midbody components were present in LECA

In non-metazoan eukaryotes, cytological studies have revealed a great diversity of structures involved in late cytokinesis, such as the plant phragmoplast and the fungal division septum [19], [26], [34]. However, cell biology data alone did not allow inferring whether these structures are homologous to the metazoan midbody (i.e., derive from an ancestral structure) or just analogous (i.e., have independent evolutionary origins) [17]. Thus, two competing hypotheses could be proposed for the origin of the midbody (Figure S1): (i) it appeared recently in the metazoan lineage after its separation from the fungal lineage or (ii) it derived from a more ancient structure that was present in the ancestor of Opisthokonta (i.e. ancestor of Metazoa and Fungi) or even in the Last Eukaryotic Common Ancestor (LECA). In the first case, the midbody would be expected to have no counterpart in non-metazoan species, whereas, in the second case, the ancestral structure from which the midbody derives could also be at the origin of the structures involved in cytokinesis in the other eukaryotic lineages. Although no proteomic data are available for those structures, phylogeny-based inference of the number of orthologues in non-metazoan lineages can be useful to discriminate whether the midbody is a metazoan-specific innovation (few orthologues would be expected to be conserved in non-metazoan lineages) or a descendant from a more ancient structure (a large set of orthologues should be present in various eukaryotic lineages). Accordingly, we analysed the orthologues found in 32 complete (or almost complete) genome sequences representative of the eukaryotic diversity, including species from most major eukaryotic lineages (Figure S1). At this step, the use of complete genome data was essential since it allowed the identification of true gene absences in the lineages studied.

**Figure 2:**
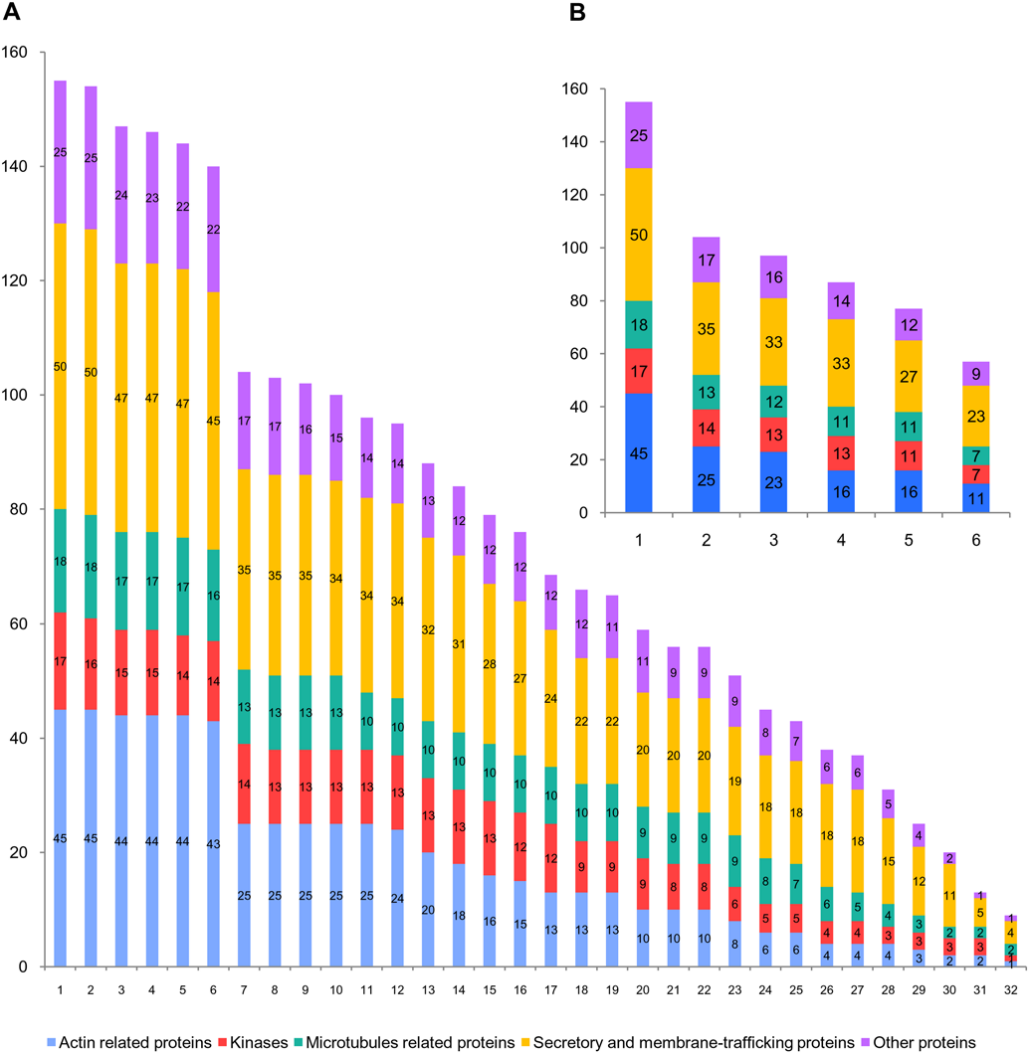
Shared orthologues of the hamster midbody among eukaryotic lineages. Number of midbody components shared (A) by an increasing number of single eukaryotic species (from at least 1 to 32) and (B) by an increasing number of eukaryotic phyla (from 1 to 6). Colours represent functional categories as in Figure 1.

The phylogenetic analysis of each of the 155 midbody proteins showed that only nine of them were shared by all the 32 eukaryotic species: actin (act1), casein kinase II (kin3), alpha tubulin (mic1), beta tubulin (mic2), G protein beta 2 (oth9), clathrin heavy chain (sec14), COPI alpha subunit (sec16), dynamin (sec19/sec20/sec21), and sec23 (sec44). This appeared to support the hypothesis of a recent metazoan origin of the midbody. However, this extremely reduced number of shared components could also reflect a poor representation of midbody components only in a few particular species or lineages. To clarify this point, we analysed the number of orthologues shared by an increasing number of eukaryotic species (i.e. how many orthologues are found in at least two eukaryotic species, three eukaryotic species, and so on). As the number of species increased, the number of shared midbody components decreased in a very regular way until an almost complete extinction (Figure 2A). This reflected regular and independent losses that occurred in all the eukaryotic species. To minimize the impact of such species-specific losses, we carried out a similar analysis but considering the number of components shared by an increasing number of phyla (Figure 2B).

In sharp contrast with the previous single-species analysis, the phylum-based comparison allowed inferring that 57 proteins were shared by the six eukaryotic phyla, i.e., six times more than the nine components shared by all 32 eukaryotic species. Although some very divergent homologues might have escaped detection, this confirmed the hypothesis that the 32 eukaryotic genomes analysed shared only a very small number of components because of independent losses that occurred at the species level. Accordingly, the number of midbody proteins inferred to have existed in the ancestors of each phylum was significantly higher than that observed in single species (Figure 1A and 1B). For example, we inferred 82 components in the ancestor of Alveolata and Heterokonta compared to a maximum of 65 detected in *Tetrahymena thermophila* and in *Phytophthora ramorum*. Similarly, we inferred that 94 orthologues were present in the ancestor of Fungi while only 71 were detected in *Saccharomyces cerevisiae* (Figure 1A and 1B). Surprisingly, the number of orthologues of midbody components found in each species was highly variable, even in species belonging to the same phylum. For example, within the Amoeboza, we identified 87 orthologues in *D. discoideum* and only 60 in its relative *Entamoeba histolytica* (Figure 1A). Similarly, within ascomycetous fungi, *Aspergillus fumigatus* contained 89 orthologues, 18 more than *S. cerevisiae*, suggesting recent losses in the latter. This suggested different tempos of component loss across species, which may be relatively high for some of them, especially in parasites such as the apicomplexan *Theileria parva* or the diplomonad *Giardia lamblia* (with only 39 and 41 orthologues identified, whereas 55 and 73 are inferred in the ancestor of Apicomplexa and in the ancestor of Excavata, respectively, Figure 1A). Surprisingly, despite the fact that we used the metazoan midbody as reference, the number of orthologues detected did not correlate with the phylogenetic distance of each phylum to the Metazoa. For example, Metazoa did not share more orthologues with the closely related Fungi than they did with the much more distant land plants (an average of 83 in both cases).

**Figure 3:**
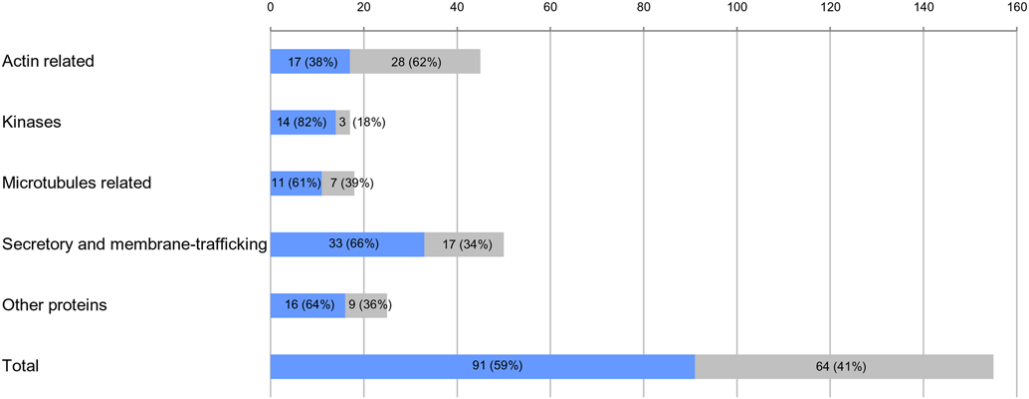
Evolutionary origin of the midbody components. Components inferred to have been present in the Last Eukaryotic Common Ancestor (LECA) are represented by blue bars, whereas those having a more recent origin (i.e. that appeared in the lineage leading from LECA to Mammalia) are represented by grey bars. Details for each functional class are provided.

Using the number of orthologues deduced for the ancestor of each eukaryotic phylum, we inferred that genes coding for at least 91 components (59%) of the mammalian midbody were already present in LECA. This implied that 56 additional midbody components originated later, in the branch leading from LECA to the ancestor of Metazoa (which likely contained 147 proteins, see above and Figure 1A). Therefore, the metazoan midbody appeared to have a dual nature, resulting from the combination of ancient (already present in LECA) and more recent components. The 91 LECA orthologues were well distributed among the five functional categories defined by Skop et al. [22] (Figure 3). In fact, we inferred that LECA possessed 82% of the kinases, 61% of the microtubule-associated proteins, 38% of the actin-associated proteins, 66% of the secretory and membrane-trafficking proteins and 64% of the proteins with other functions (Figure 3). Similar trends were observed for present-day species. The only exception concerned the actin-associated proteins (Figure 1A), for which dramatic losses have occurred in several unrelated lineages, such as Apicomplexa or the diplomonad *G. lamblia* (with only 4–6 and 2 out of 45 components detected, respectively, Figure 1A). This may reflect a less important role of actin in the cytokinesis of these eukaryotes. As a matter of fact, microtubules instead of actin filaments have been shown to drive daughter cell budding and cell division in Apicomplexa [35] whereas in the case of *G. lamblia*, a remarkable acceleration of the evolutionary rate in its actin sequence was observed (Figure S2). These modifications involving actin may have led to an evolutionary convergence in these lineages through the loss (or the replacement) of most actin-associated proteins.

### Functional conservation in the midbody orthologues

**Figure 4:**
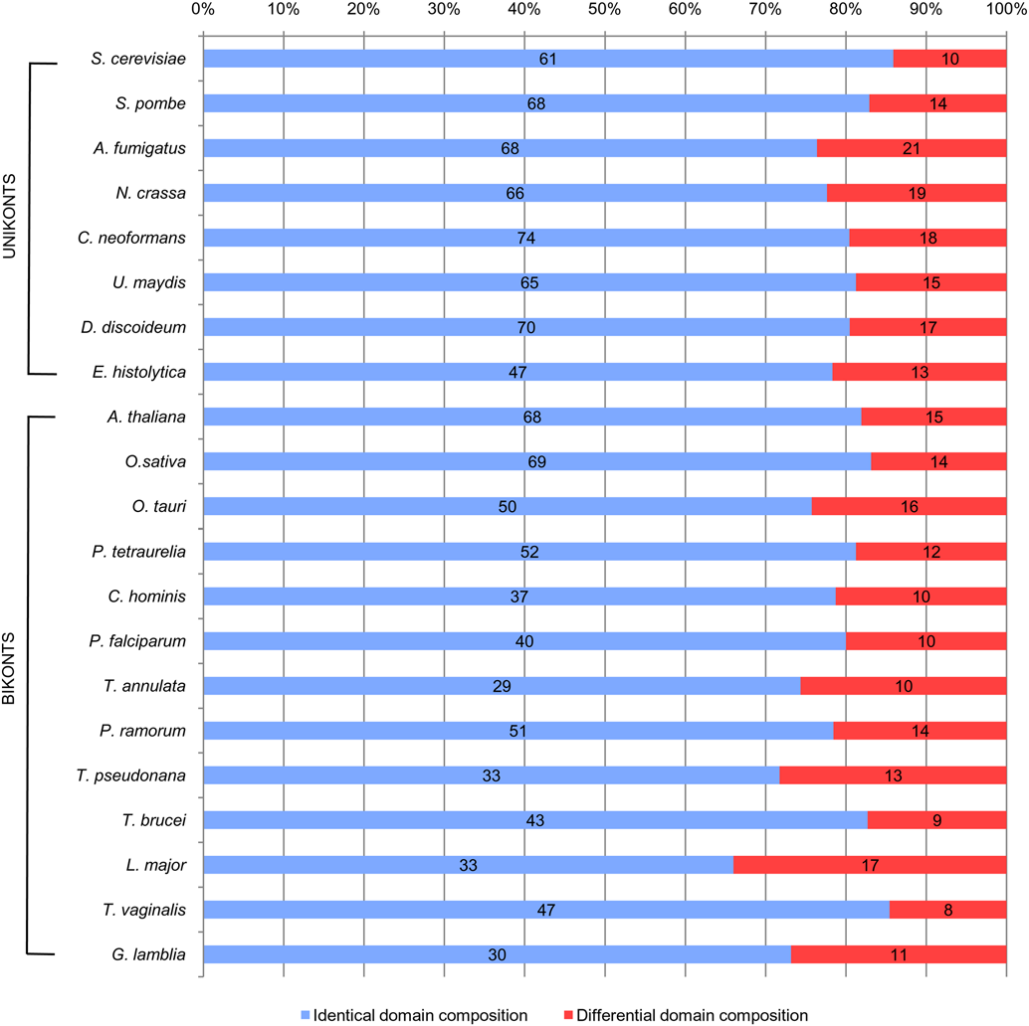
Domain conservation of the orthologues of the hamster midbody among eukaryotic lineages. Conservation of functional domains composition among mammalian components, and their orthologues in fungi (*A. fumigatus, Neurospora crassa, Cryptococcus neoformans, Ustilago maydis, Schizosaccharomyces pombe* and *S. cerevisiae*), amoebozoa (*D. discoideum* and *E. histolytica*), green plants and algae (*A. thaliana*, *Oryza sativa* and *Ostreococcus tauri*), alveolates (*Paramecium tetraurelia Cryptosporidium hominis, Plasmodium falciparum,* and *Theileria annulata*), heterokonts (*P. ramorum* and *T. pseudonana*) and excavates (*T. brucei, Leishmania major, G. lamblia* and *Trichomonas vaginalis*). Orthologues in these 21 non-animal species having identical functional domains composition to their mammalian counterparts are represented by blue bars, whereas those having a different functional domain composition are represented by red bars.

To get more insight in the functional characterisation of the orthologues of non-metazoan lineages and those inferred in LECA, we studied their functional domain organisation in representatives of each eukaryotic phylum (Figure 4). We found that 66% up to 86% (average of 79%) of the orthologues in these non-metazoan species had functional domain compositions identical to those of their mammalian counterparts (Figure 4 and Tables S2 and S3). Although available functional data are scarce for non-metazoan lineages, this striking conservation across very distant eukaryotic lineages strongly suggested that the non-metazoan orthologues have the same molecular functions than the mammalian ones and, therefore, may have similar cellular functions. To test this hypothesis, we carried out a survey of the *Saccharomyces* Genome Database (SGD, http://www.yeastgenome.org/) that showed that 31 of the 71 yeast orthologues (44%) have been characterised as involved in cytokinesis or cell division, and 55 (77%) have been found to interact with at least one of the other 71 (Table S4). These observations strongly suggested that, as in Metazoa, most of the yeast orthologues act together and are involved in cell division, a characteristic likely inherited from the last common ancestor of Fungi and Metazoa. In Amoebozoa, functional data are too scarce to make this kind of analysis. However, among the high number of orthologues detected in the amoeba *D. discoideum* (at least 87), 70 have the same domain organisation of their metazoan orthologues, which correlated well with the description in this species of a cytokinesis structure that presents clear similarities with the animal midbody [20], [24]. This suggests that this structure, the septum of Fungi and the metazoan midbody most likely derive from an ancestral structure already present in the ancestor of Unikonts.

These results are in agreement with a recent comparative study of 24 functionally characterised components of the midbody of various animals, the division septum of two fungi and the phragmoplast of plants, which suggested that most of them are homologous in these three distant groups, tracing back their origin to LECA [17]. Among those 24 components, we detected ten in the proteome of the hamster midbody (Table S5), and confirmed that at least four of them were true orthologues in the three groups (animals, fungi and plants), and eight were orthologues at least in two of them. For example, kin1 (called AIM-1 in mammals and AIR-2 in *C. elegans*) is essential for normal central spindle and cleavage furrow formation, and for the ultimate separation of the two daughter cells in Metazoa [36], [37]. Its orthologues AtAurora 1 and 2 of *A. thaliana* and Ipl1p of *S. cerevisiae* have been shown to be associated to the phragmoplast and necessary to ensure appropriate positioning and assembly of the new cell wall in plants [38], and important for chromosome segregation in yeast [39], respectively. An additional evolutionary link between the midbody and the plant phragmoplast comes from the fact that the phragmoplast grows predominantly by the fusion of Golgi-derived vesicles [27] and it has been shown that 38 mammalian midbody components are Golgi-associated proteins [22].

In summary, our results support that (i) nearly 60% of the present-day mammalian midbody components were present in LECA and were well distributed in all the five functional categories, (ii) an average of 79% of the orthologues identified in a variety of unikonts and bikonts displayed a high conservation of functional domain composition, indicating a similar molecular function, (iii) most of these orthologues interact in yeast and are likely involved in cytokinesis, and (iv) a number of these orthologues are part of the plant phragmoplast. All these four lines of evidence suggested that these structures derive from a much more ancient one dating back to LECA. This ancestral structure may have been composed of at least 90 interacting components and was already involved in cytokinesis. It is important to note here that the estimation of that ancestral number of components was likely underestimated since our analysis was based on the only proteome available (the hamster midbody), preventing the detection of all the components that have been lost specifically in the lineage leading from LECA to Mammalia. The characterisation of proteomes of equivalent structures in other eukaryotic lineages (especially in bikonts, such as the plant phragmoplast) will allow confirming the relationship between these structures, but also having a better estimation of the number of ancestral components and a clearer picture of the complexity of the ancestral structure that was present in LECA. Finally, the future availability of additional data from non-metazoan lineages will highlight the gains or replacements that occurred in each lineage. For example, it is expected that similar gains as the 56 new proteins recruited in the lineage leading from LECA to Metazoa also occurred in other lineages. The identification of these gains may help to explain the important differences observed in the structures involved in late cytokinesis in the different eukaryotic groups.

### Testing the hypothesis of a prokaryotic origin

Since a complex multi-protein structure involved in cytokinesis most likely existed in LECA, it would be interesting to test if it derived from an even older structure that would have been present in prokaryotic ancestors. To address this question, we searched for homologues of the 155 hamster midbody components in all available complete prokaryotic genomes (730 genomes). We detected homologues for only nine: Actin (act1), ADP-Ribosylation factor-like 1 (sec2), Flotillin 1 (sec26), Gmx33/Golph3 (sec29), NSF (sec36), Rack 1 (sec42), alpha-tubulin (mic1), beta-tubulin (mic2), Novel/CGI-49 (oth19). Nevertheless, phylogenetic analyses revealed that four of them (Flotillin 1, Gmx33/Golph3, and alpha and beta-tubulin) likely resulted from horizontal gene transfer between eukaryotes and prokaryotes (Figures S3, S4, S5, S6). Therefore, only five of the midbody components inferred to have existed in LECA were probably present also in prokaryotic ancestors. Although this was a minimal set since, once again, very divergent prokaryotic homologues might have escaped detection, this small number indicated that nearly all the proteins present in LECA appeared after the emergence of the eukaryotic lineage.

**Figure 5:**
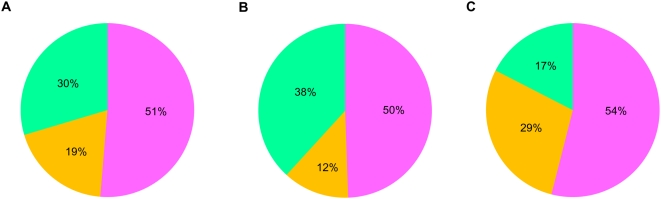
Functional domain composition of the mammalian midbody proteins. Pink sections represent proteins composed only of eukaryotic-specific functional domains (i.e. they have no homologues in prokaryotes, meaning that they probably appeared after the divergence of eukaryotes and prokaryotes); green sections represent proteins exclusively composed of domains shared by prokaryotes and eukaryotes (i.e. suggesting that these domains may have a more ancient origin since they may have arisen before eukaryotic divergence); yellow sections represent proteins composed by a combination of both types of domains. (A) Domain composition of 152 components of the mammalian midbody having detectable functional domains. The three remaining proteins have no functional domain detected above threshold. (B) Domain composition of 88 components inferred to be present in LECA having detectable functional domains. (C) Domain composition of the 64 Unikont-specific components.

Such a small number of orthologues detected in prokaryotes does not mean that all midbody components completely originated *de novo* in eukaryotes. Indeed, the analysis of the protein domain composition of the hamster midbody proteins showed that only half of them were composed of domains that are specific to eukaryotes (i.e. domains that originated after the divergence of eukaryotes and prokaryotes) (Figure 5A). Interestingly, this proportion did not change when the analysis was restricted to the 91 components already present in LECA or to the 64 components of more recent origin (i.e., those that originated in the lineage leading from LECA to the present-day mammals) (50% and 54%, respectively, Figure 5B and 5C). By contrast, the proportion of components composed exclusively of domains that are also found in prokaryotes (i.e., ancient domains probably present in the ancestor of eukaryotes and prokaryotes) gone down from 38% to 17% (Figure 5B and 5C), whereas the proportion of components composed of a combination of both types of domains showed an opposite trend, going up from 12% to 29% (Figure 5B and 5C, respectively). This implied that only half of the hamster midbody proteins were actually *de novo* eukaryotic innovations whereas the other half derived from tinkering of more ancient protein domains, sometimes in combination with recent ones.

### Conclusion

Although still scarce, some phylogenomic studies of EMC involved in major eukaryotic processes have suggested that LECA was already a highly complex and modern-like eukaryotic cell. In fact, it likely had a nuclear pore complex [13], [14] and, therefore, a nuclear membrane [14], a spliceosome thought to be similar in complexity to contemporary ones [15], and the basic architecture of the vesicle trafficking system [40], [41]. The idea of a LECA with complex intracellular structures is in agreement with the estimation of the number of genes present in this ancestral organism (more than 4100 [42], suggesting that it had a rather large genome. Our analysis provided additional evidence for the hypothesis of a nearly-modern eukaryotic LECA, because we showed that it likely possessed a large multiprotein complex involved in the last steps of cytokinesis, from which the various eukaryotic present-day related structures derived. The future availability of genomic and proteomic data for diverse eukaryotic lineages will allow generalizing phylogenomic studies to other eukaryotic cellular structures. These analyses are expected to provide a more precise picture of LECA, as well as of the evolution of the different eukaryotic lineages. Finally, besides evolutionary inferences, an important aspect of this kind of work is the identification of components of cellular complexes in eukaryotic lineages for which data are inexistent or sparse, thus providing candidates for experimental tests in these groups.

## Materials and Methods

### Data set construction

Homologues of each of the 160 proteins identified in the hamster midbody proteome were retrieved from the *nr* database at the NCBI using the BLASTp program version 2.2.18 [43]. Additional BLASTp searches were performed using various seeds in order to retrieve divergent homologues that escaped detection in the first search. This multiple-step BLASTp approach was used to ensure that each gene family investigated was exhaustively sampled. Homologues from *Thalassiosira pseudonana* and *Phytophthora ramorum* were retrieved from the eukaryotic ongoing genome projects at the NCBI using the tBLASTn [43]. For each of the 160 assembled datasets, multiple alignments were done with ClustalW version 1.83 [44], T-COFFEE version 4.45 [45] and MUSCLE 3.6 [46]. The best alignment (in terms of maximal length and minimal number of gaps) was kept for further analyses. All the alignments were edited and manually refined using the ED program from the MUST package [47]. Regions where homology between sites was doubtful were manually removed from the datasets before phylogenetic analyses using the program NET from the MUST package. All datasets are available on request from LE or CB.

### Phylogenetic analyses

For each dataset, Maximum Likelihood (ML) phylogenetic trees were computed with PHYML using the JTT model and a gamma correction to take into account the heterogeneity of evolutionary rates across sites (4 discrete classes of sites, an estimated alpha parameter and an estimated proportion of invariable sites) [48]. The robustness of each branch was estimated by the non-parametric bootstrap procedure implemented in PHYML (100 replicates of the original dataset and the same parameters). We carried out a first round of phylogenetic analyses to identify the orthologues of each mammalian midbody component. Based on these phylogenies, we selected the orthologues present in 32 eukaryotes for which complete (or nearly complete) genome sequences were available and representative of the diversity of this domain (Figure S1). Then, a second round of phylogenetic analyses was performed on these orthologues.

### Inference of the number of components present at each node of the eukaryotic phylogeny

We applied a simple presence/absence parsimony criterion minimising the occurrence of horizontal gene transfers between eukaryotic lineages for inference. This implies that the presence of an orthologue of a mammalian midbody component in one representative of a non-mammalian lineage was interpreted as the existence of the component in their last common ancestor. The use of more stringent criteria (such as the presence of orthologues in representatives of at least two or three non mammalian-lineage) did not significantly change the number of components inferred at each node (not shown).

### Analysis of functional domain conservation

The analysis of functional domains was carried out using the HMMER package [49]. First, we identified the functional domain composition of all the eukaryotic orthologues of the components of the mammalian midbody by performing hmmpfam searches against a local database of HMM profiles (pfam database [50] – version 21.0). HMM profiles having e-values lower than 0.1 were considered as significant if the corresponding domains did not overlap in the protein sequences.

### Search for functional domains shared with prokaryotes

The hmmfetch program was used to extract each significant HMM profile corresponding to functional domains previously identified in mammalian midbody components. A survey for sequences matching these profiles on a local database of protein sequences from the 730 complete prokaryotic genomes available in june 2008 at the NCBI was done using the hmmsearch program.

## Supporting Information

Figure S1 Phylogenetic tree of Eukaryotes. A consensus phylogeny of eukaryotes showing the phylogenetic position of the 32 eukaryotic representatives used in our study. The Last Eukaryotic Common Ancestor is indicated by a grey dot, whereas red stars indicate alternative positions for the origin of the midbody: red star "1" indicates a recent origin of the midbody (i.e. outbreak in the metazoan lineage), red stars "2" and "3" point to two possibilities for an ancient origin of the midbody (i.e. emergence before the ancestor of opisthonkonts or before LECA).
(3.04 MB TIF)

Figure S2 Phylogeny of actin. Maximum Likelihood (ML) tree of the actin homologues present in the 32 eukaryotic lineages studied (360 positions analysed). Numbers at nodes represent Bootstrap Values (for clarity only those greater than 50% are shown). The scale bar represents the average number of substitutions per site.
(4.68 MB TIF)

Figure S3 Phylogeny of flotillin 1. ML tree of the flotillin 1 (sec26) homologues present in the 32 eukaryotic lineages studied and in prokaryotes (186 positions analysed). Numbers at nodes represent Bootstrap Values (for clarity only those greater than 50% are shown). The scale bar represents the average number of substitutions per site.
(2.85 MB TIF)

Figure S4 Phylogeny of gmx33/golph3. ML tree of the gmx33/golph3 (sec29) homologues present in the 32 eukaryotic lineages studied and in prokaryotes (105 positions analysed). Numbers at nodes represent Bootstrap Values (for clarity only those greater than 50% are shown). The scale bar represents the average number of substitutions per site.
(2.24 MB TIF)

Figure S5 Phylogeny of alpha-tubulin. ML tree of the alpha-tubulin homologues present in the 32 eukaryotic lineages and in prokaryotes (318 positions analysed). Numbers at nodes represent Bootstrap Values (for clarity only those greater than 50% are shown). The scale bar represents the average number of substitutions per site.
(4.43 MB TIF)

Figure S6 Phylogeny of beta-tubulin. ML tree of the beta-tubulin homologues present in the 32 eukaryotic lineages and in prokaryotes (345 positions analysed). Numbers at nodes represent Bootstrap Values (for clarity only those greater than 50% are shown). The scale bar represents the average number of substitutions per site.
(3.98 MB TIF)

Table S1 Proteome of the mammalian midbody. The proteins are classified according to the five functional categories defined by Skop et al. (actin associated proteins, kinases, microtubules associated proteins, secretory and membrane trafficking associated proteins, and other) [22]. The name and the Genbank accession number of each protein are provided. The components inferred to have been present in the ancestor of Metazoa, Fungi, Amoebozoa, Plantae, Alveolata, Heterokonta, Excavata, as well as those present in LECA are indicated by an "x". As act33 and kin6 were discarded from our phylogenetic analyses, their presence in the different ancestors was not determined (ND).
(0.13 MB PDF)

Table S2 Functional domain composition of the mammalian midbody proteins. This table shows the domain composition of each of the 155 components of the hamster midbody. The name and the PFAM accession number of each domain are provided.
(0.06 MB PDF)

Table S3 Comparison of the functional domain composition of mammalian midbody proteins with their non metazoan eukaryotic orthologues. Comparison with the domain composition of the orthologues found in Aspergillus fumigatus, Neurospora crassa, Cryptococcus neoformans, Ustilago maydis, Saccharomyces cerevisiae, Schizosaccharomyces pombe, Dictyostelium. Discoideum, Entamoeba histolytica, Arabidopsis thaliana, Oryza sativa, Ostreococcus tauri, Paramecium tetraurelia, Cryptosporidium hominis, Plasmodium falciparum, Theileria annulata, Phytophtora Ramorum, Thalassiosira pseudonana, Trypanosoma brucei, Leishmania major, Giardia lamblia and Trichomonas vaginalis. Asterisks indicate orthologues having the same domain composition as their mammalian counterparts; dollars designate orthologues displaying at least one difference with their mammalian counterparts; whereas "no" indicate that no orthologues are available.
(0.27 MB PDF)

Table S4 Physical interactions between orthologues of the mammalian midbody components found in Saccharomyces cerevisiae. Interaction data was taken from a survey of the Saccharomyces Genome Database (SGD, http://www.yeastgenome.org/). For each of the 71 yeast orthologues, the standard names of the orthologues with which it interacts are indicated. Components marked by an asterisk are annotated as involved in cytokinesis, or in a biological process related to cell division.
(0.04 MB PDF)

Table S5 Components of the midbody, fungal septum division and phragmoplast studied by Otegui et al. and their relations of orthology. Grey columns correspond to proteins studied by Otegui et al. in mammals, Saccharomyces cerevisiae, Schizosaccharomyces pombe and plants [17]. Orthologues of these components present in the hamster midbody proteome are indicated by a cross. Dollars indicate paralogues of components of the hamster midbody.
(0.04 MB PDF)
