# Supplementary figures and images for: A Complex Cell Division Machinery Was Present in the Last Common Ancestor of Eukaryotes
Source: PLoS One. 2009 Apr 7;4(4):e5021. doi: 10.1371/journal.pone.0005021 (PMC2661371; doi:10.1371/journal.pone.0005021)

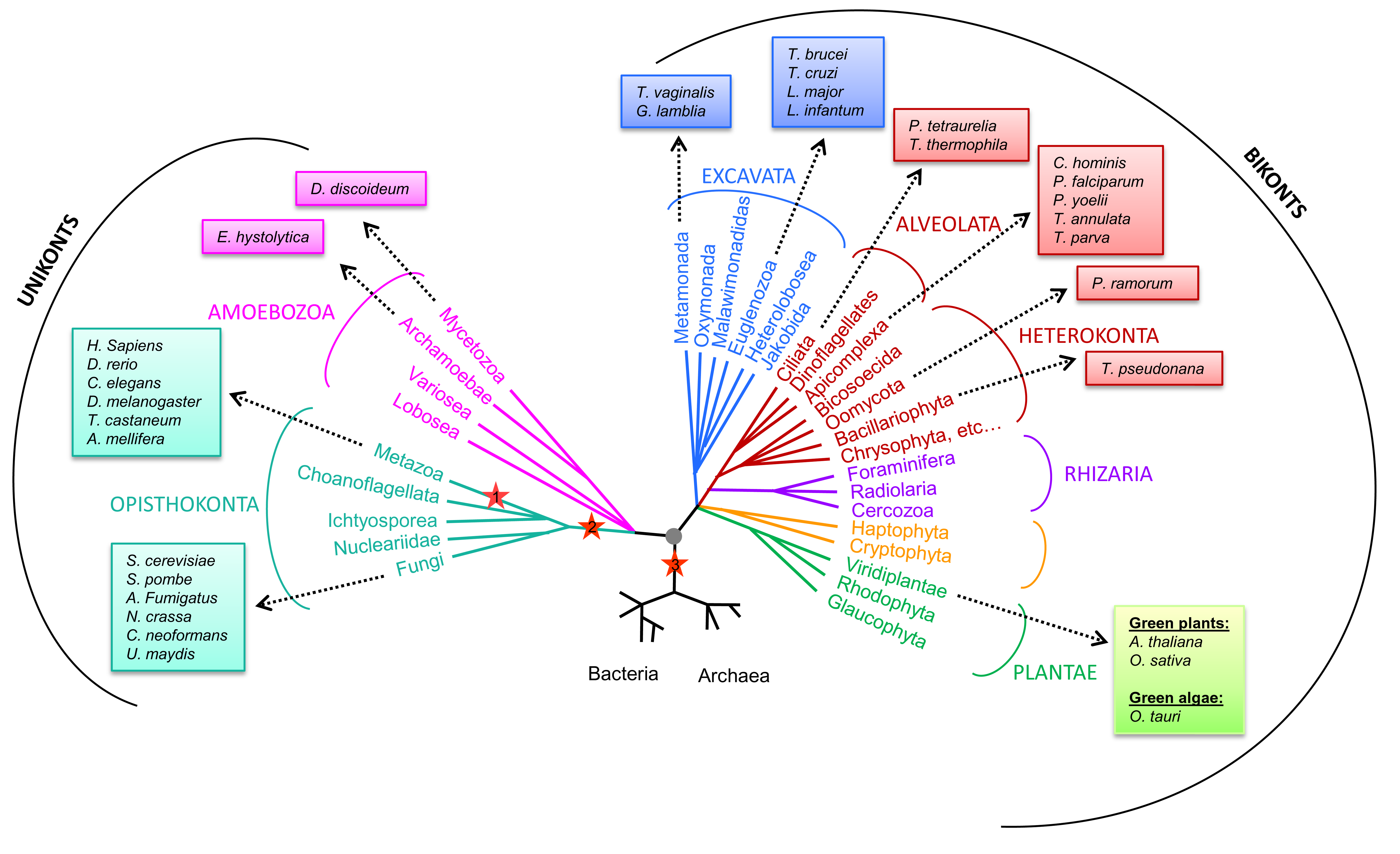

Supplement: Figure S1 — Phylogenetic tree of Eukaryotes. A consensus phylogeny of eukaryotes showing the phylogenetic position of the 32 eukaryotic representatives used in our study. The Last Eukaryotic Common Ancestor is indicated by a grey dot, whereas red stars indicate alternative positions for the origin of the midbody: red star "1" indicates a recent origin of the midbody (i.e. outbreak in the metazoan lineage), red stars "2" and "3" point to two possibilities for an ancient origin of the midbody (i.e. emergence before the ancestor of opisthonkonts or before LECA). (3.04 MB TIF) [file pone.0005021.s001.tif]

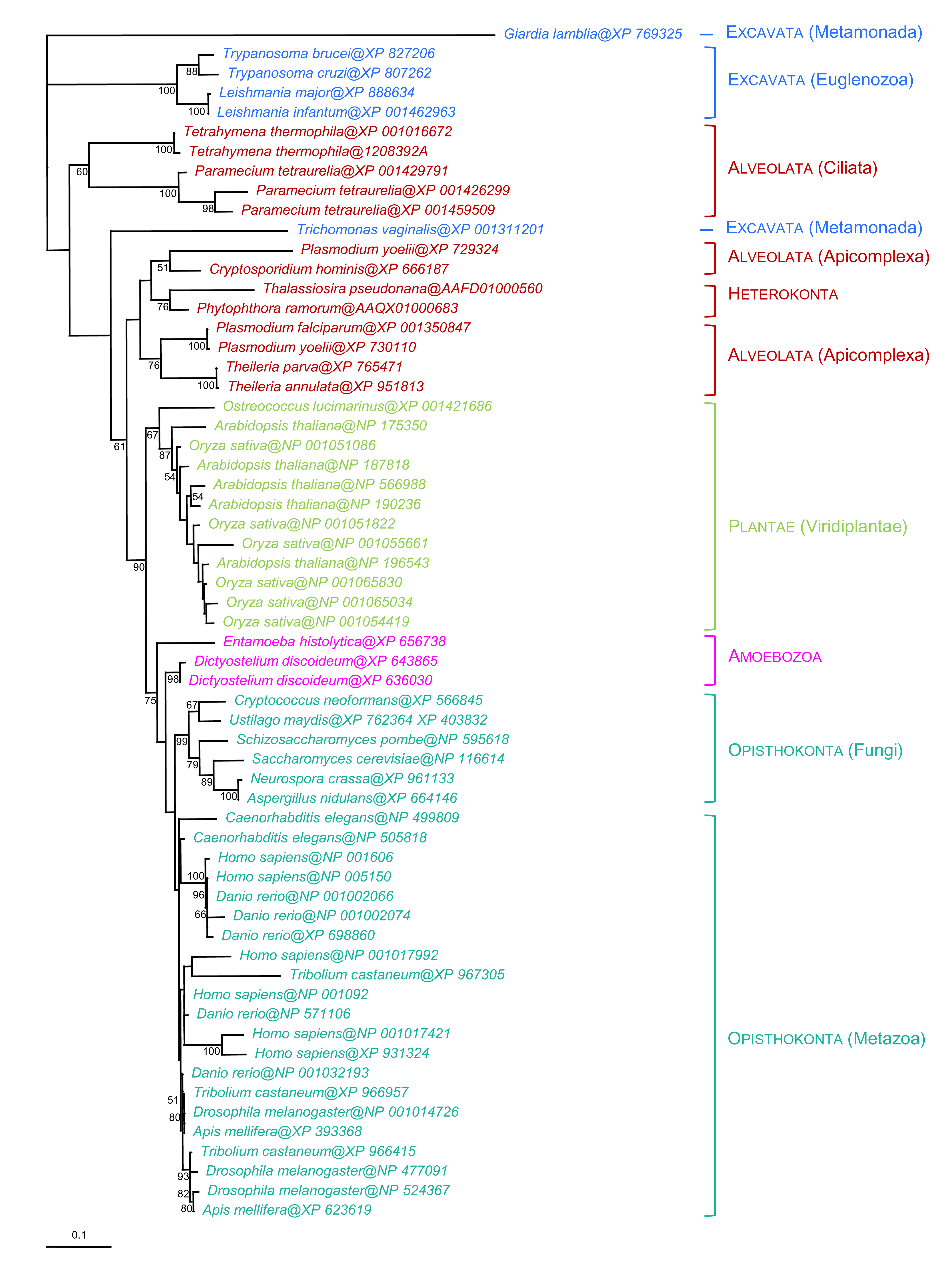

Supplement: Figure S2 — Phylogeny of actin. Maximum Likelihood (ML) tree of the actin homologues present in the 32 eukaryotic lineages studied (360 positions analysed). Numbers at nodes represent Bootstrap Values (for clarity only those greater than 50% are shown). The scale bar represents the average number of substitutions per site. (4.68 MB TIF) [file pone.0005021.s002.tif]

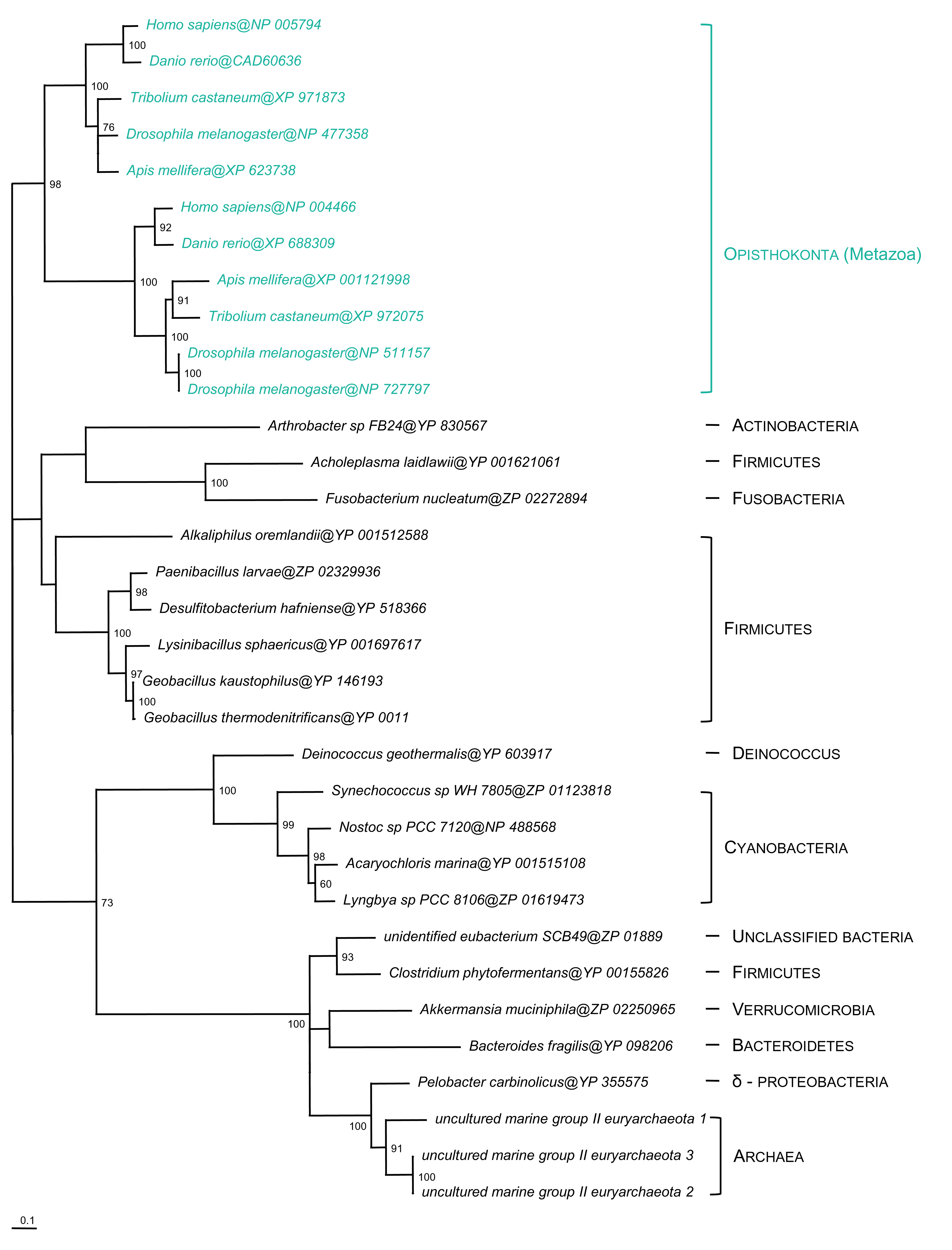

Supplement: Figure S3 — Phylogeny of flotillin 1. ML tree of the flotillin 1 (sec26) homologues present in the 32 eukaryotic lineages studied and in prokaryotes (186 positions analysed). Numbers at nodes represent Bootstrap Values (for clarity only those greater than 50% are shown). The scale bar represents the average number of substitutions per site. (2.85 MB TIF) [file pone.0005021.s003.tif]

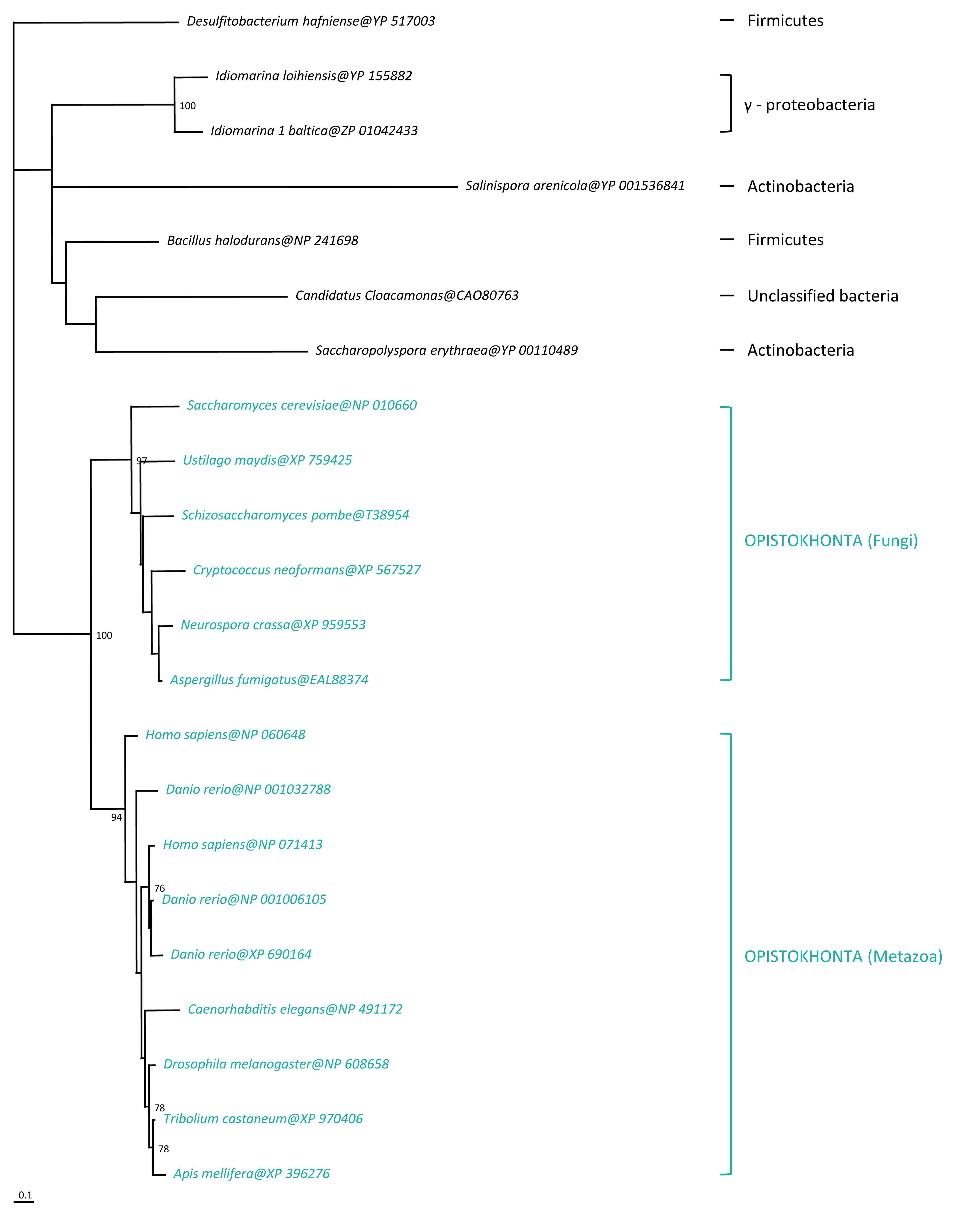

Supplement: Figure S4 — Phylogeny of gmx33/golph3. ML tree of the gmx33/golph3 (sec29) homologues present in the 32 eukaryotic lineages studied and in prokaryotes (105 positions analysed). Numbers at nodes represent Bootstrap Values (for clarity only those greater than 50% are shown). The scale bar represents the average number of substitutions per site. (2.24 MB TIF) [file pone.0005021.s004.tif]

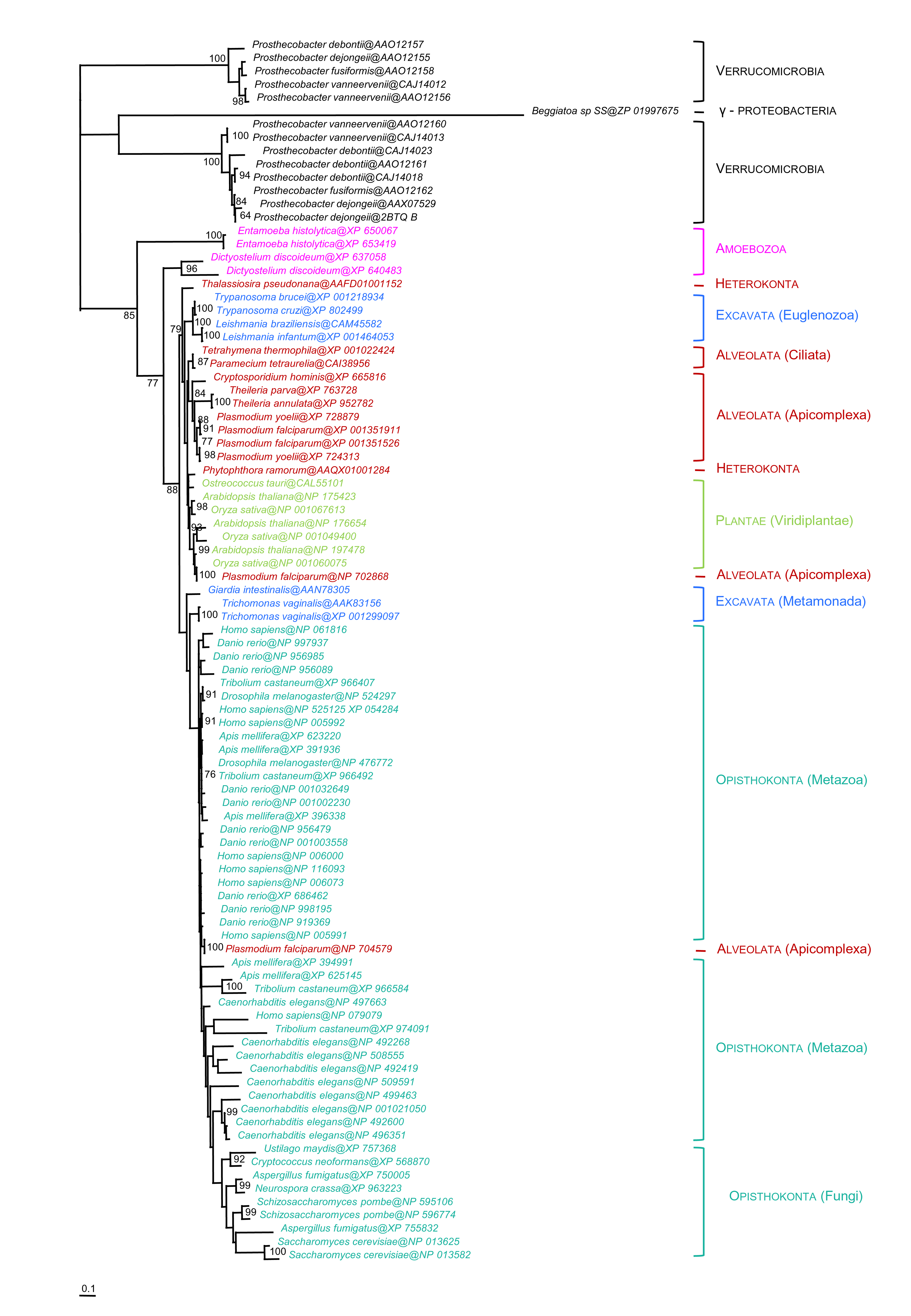

Supplement: Figure S5 — Phylogeny of alpha-tubulin. ML tree of the alpha-tubulin homologues present in the 32 eukaryotic lineages and in prokaryotes (318 positions analysed). Numbers at nodes represent Bootstrap Values (for clarity only those greater than 50% are shown). The scale bar represents the average number of substitutions per site. (4.43 MB TIF) [file pone.0005021.s005.tif]

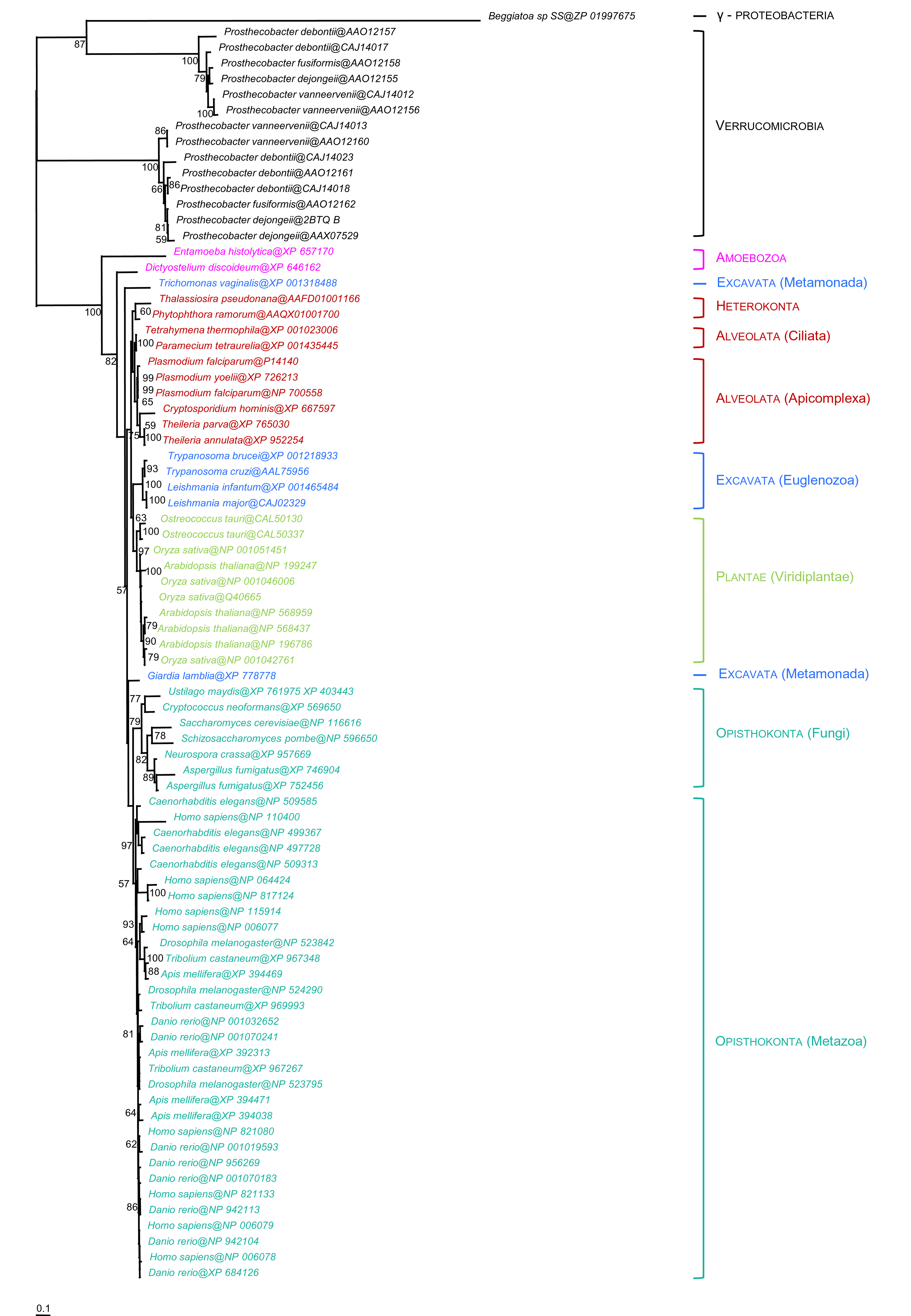

Supplement: Figure S6 — Phylogeny of beta-tubulin. ML tree of the beta-tubulin homologues present in the 32 eukaryotic lineages and in prokaryotes (345 positions analysed). Numbers at nodes represent Bootstrap Values (for clarity only those greater than 50% are shown). The scale bar represents the average number of substitutions per site. (3.98 MB TIF) [file pone.0005021.s006.tif]
