# Supplementary material for: A Complex Cell Division Machinery Was Present in the Last Common Ancestor of Eukaryotes
Source: PLoS One. 2009 Apr 7;4(4):e5021. doi: 10.1371/journal.pone.0005021 (PMC2661371; doi:10.1371/journal.pone.0005021)
Supplement: Table S1 — Proteome of the mammalian midbody. The proteins are classified according to the five functional categories defined by Skop et al. (actin associated proteins, kinases, microtubules associated proteins, secretory and membrane trafficking associated proteins, and other) [22]. The name and the Genbank accession number of each protein are provided. The components inferred to have been present in the ancestor of Metazoa, Fungi, Amoebozoa, Plantae, Alveolata, Heterokonta, Excavata, as well as those present in LECA are indicated by an "x". As act33 and kin6 were discarded from our phylogenetic analyses, their presence in the different ancestors was not determined (ND). (0.13 MB PDF) [file pone.0005021.s007.pdf]

Supplementary Information Table 1. Proteome of the mammalian midbody.

Short name	Mammalian Protein	Accession number	Fonctional group	Present in the ancestor of							
				Eukaryotes	Metazoa	Fungi	Amoebozoa	Plantae	Alevolata	Heterokonta	Excavata
act1	ACTIN	113272	Actin Associated	x	x	x	x	x	x	x	x
act2	ALPHA II SPECTRIN	1495198	Actin Associated		x						
act3	ARP2	3121762	Actin Associated	x	x	x	x	x	x	x	x
act4	ARP2/3 P41 SUBUNIT	3121763	Actin Associated	x	x	x	x	x	x	x	x
act5	ARP3	416579	Actin Associated	x	x	x	x	x	x	x	x
act6	TUFTELIN-INTERACTIN ASSOCIATED G PROTEIN 33/SHORT STOP HOMOLOG	10190660	Actin Associated	x	x	x	x	x		x	
act7	COFILIN	105664	Actin Associated	x	x	x	x	x	x	x	x
act8	MYOSIN I HC	1083723	Actin Associated	x	x	x	x	x	x	x	x
act9	MYOSIN II NONMUSCLE	10879497	Actin Associated		x	x	x				
act10	IQGAP1	1170586	Actin Associated		x	x	x				
act11	MYOSIN LC	127144	Actin Associated		x	x	x				
act12	F-ACTIN ASSOCIATED CAPPING PROTEIN BETA SUBUNIT/CAPZ	1345667	Actin Associated	x	x	x	x	x	x	x	x
act13	PINCH	1346721	Actin Associated		x						
act14	VIMENTIN	138536	Actin Associated		x						
act15	ZO-1/PLAKOGLOBIN	1709649	Actin Associated		x						
act16	PLECTIN	1709655	Actin Associated		x						
act17	EZRIN	2119262	Actin Associated		x						
act18	DREBRIN 1	2143704	Actin Associated		x	x	x				
act19	ARP2/3 P21 SUBUNIT	2209347	Actin Associated	x	x	x	x	x	x	x	x
act20	MOESIN	2218139	Actin Associated		x						
act21	TALIN	227256	Actin Associated		x						
act22	SEPTIN 6	2500770	Actin Associated		x	x					
act23	T-PLASTIN/FIMBRIN	2780868	Actin Associated	x	x	x	x	x	x	x	x
act24	ECT2 RHO GEF/PEBBLE HOMOLOG	293332	Actin Associated		x						
act25	BAND 4.1	3064263	Actin Associated		x						
act26	FILAMIN/ABP-278	3282771	Actin Associated		x						
act27	BETA SPECTRIN	448251	Actin Associated		x						
act28	ALPHA ACTININ 4	4826639	Actin Associated	x	x	x	x			x	x
act29	ARP2/3 P34 SUBUNIT	5031599	Actin Associated	x	x	x	x	x	x	x	x
act30	CALPONIN	584956	Actin Associated		x						
act31	DIAPHANOUS HOMOLOG 1	6225268	Actin Associated		x	x	x				
act32	CDC42	6531681	Actin Associated		x	x					
act33	ANKYRIN B/ANK2	6634025	Actin Associated	ND	x	ND	ND	ND	ND	ND	ND
act34	RADIXIN	6677699	Actin Associated		x						
act35	VINCULIN	6724323	Actin Associated		x						
act36	CORONIN	6753496	Actin Associated	x	x	x	x		x	x	x
act37	SEPTIN/NEDD5/CDCREL-1	6754816	Actin Associated	x	x	x			x		
act38	PROFILIN	6755040	Actin Associated	x	x	x	x	x	x		x
act39	TROPOMYOSIN	7513846	Actin Associated	x	x	x		x	x	x	x
act40	SMOOTHELIN B	7547259	Actin Associated								
act41	ZO-2	7549795	Actin Associated		x						
act42	MYOSIN REGULATORY LIGHT CHAIN 2-B	8393781	Actin Associated		x	x	x				
act43	ENIGMA	8515740	Actin Associated		x						
act44	BAF53A /ACTIN ASSOCIATED-LIKE 6	9789893	Actin Associated	x	x	x	x	x	x		
act45	NADRIN/RICH1RHOGAP	9971185	Actin Associated		x						
act46	KEAP1	31543042	Actin Associated		x						
kin1	AURORA KINASE B	2979628	Kinases	x	x	x	x	x	x	x	x
kin2	CAM KINASE II	92035	Kinases	x	x	x	x	x	x	x	x
kin3	CASEIN KINASE II	89457	Kinases	x	x	x	x	x	x	x	x
kin4	CDC2 KINASE	1082288	Kinases	x	x	x	x	x	x	x	
kin5	CDC28 PROTEIN KINASE 2	4502859	Kinases	x	x	x	x	x	x	x	x
kin6	CDK4	2209290	Kinases	ND	x	ND	ND	ND	ND	ND	ND
kin7	CITRON-K KINASE	3360514	Kinases								
kin8	MAP KINASE KINASE	4200234	Kinases	x	x				x		
kin9	N-TERMINAL KINASE LIKE PROTEIN 105-KDA	10442581	Kinases	x	x	x	x	x	x		
kin10	NUCLEOPHOSMIN	10835063	Kinases								
kin11	POLO-LIKE KINASE	6755104	Kinases	x	x	x	x	x	x	x	x
kin12	PP1A	7305405	Kinases	x	x	x	x	x	x		x
kin13	PP2A	107304	Kinases	x	x	x	x	x			
kin14	PP6C	2499733	Kinases	x	x	x	x	x	x	x	x
kin15	RAS SUPPRESSOR PROTEIN/RSP1	6677825	Kinases	x	x		x	x			x
kin16	ROCK2	6677761	Kinases	x	x		x	x	x		x
kin17	SERINE THREONINE KINASE 24/STE20P HOMOLOG	2582413	Kinases	x	x	x	x	x			x
kin18	STE20-RELATED KINASE SMAK	4741823	Kinases		x						
mic1	ALPHA-TUBULIN	3420929	Microtubule associated	x	x	x	x	x	x	x	x
mic2	BETA-TUBULIN	2119275	Microtubule associated	x	x	x	x	x	x	x	x
mic3	BIMC KINESIN/EG5	4160556	Microtubule associated	x	x	x	x	x	x	x	x
mic4	CLIP-170	88551	Microtubule associated		x						
mic5	DYNACTIN ASSOCIATED/P150-GLUED	6226857	Microtubule associated		x	x					
mic6	DYNAMITIN/DYNACTIN ASSOCIATED COMPLEX 50KD SUBUNIT	5453629	Microtubule associated		x	x	x				
mic7	DYNEIN HC	2224591	Microtubule associated	x	x	x	x		x	x	x
mic8	EB1	6912494	Microtubule associated	x	x	x	x	x	x	x	x
mic9	GAMMA TUBULIN COMPLEX PROTEIN 2	5729840	Microtubule associated	x	x	x	x	x	x	x	x
mic10	KIF4	5802957	Microtubule associated	x	x	x		x	x		x
mic11	KIFC1/KAR3P HOMOLOG	4050097	Microtubule associated	x	x	x	x	x	x		x
mic12	KINESIN HEAVY CHAIN	2119280	Microtubule associated	x	x	x	x	x	x	x	x
mic13	KINESIN LIGHT CHAIN	8101107	Microtubule associated		x						
mic14	MAP 1B	5174525	Microtubule associated								
mic15	MCAK/MITOTIC CENTROMERE ASSOCIATED KINESIN	5803082	Microtubule associated	x	x		x	x	x	x	x
mic16	MKLP1	6754472	Microtubule associated		x						
mic17	ORBIT/CLASP1	7513045	Microtubule associated		x						
mic18	TOG /XMAP215 HOMOLOG	2136282	Microtubule associated	x	x	x	x	x	x		
oth1	BUB3	7387554	Other	x	x	x	x	x	x	x	x

oth2	CALCYCLIN BINDING PROTEIN	7656952	Other	x	x		x	x	x	x	x
oth3	CALMODULIN	1710819	Other								
oth4	CDC16	1362769	Other	x	x	x	x	x	x		x
oth5	CDC20	4323528	Other	x	x	x	x	x	x	x	x
oth6	CDK INHIBITOR 1B	4757962	Other		x						
oth7	CULLIN 1	2493906	Other	x	x	x	x	x	x	x	x
oth8	ENHANCER OF RUDIMENTARY	4758302	Other	x	x		x	x	x	x	
oth9	G PROTEIN BETA2	121009	Other	x	x	x	x	x	x	x	x
oth10	GAS-1/GROWTH ARREST SPECIFIC-1	6679941	Other		x						
oth11	GTP-BINDING REGULATORY PROTEIN GI ALPHA-2 CHAIN	4218034	Other	x	x	x	x	x		x	x
oth12	LAMIN A/C	125962	Other		x						
oth13	LAMIN B1	125953	Other		x						
oth14	MAGO-NASHI PROTEIN	6754616	Other	x	x	x	x	x	x	x	x
oth15	MEK BINDING PARTNER 1	9910452	Other	x	x		x			x	x
oth16	NEDD4 UBIQUITIN LIGASE	2144012	Other		x	x					
oth17	NOGGIN	7110675	Other		x						
oth18	NOVEL/AAC52863	1644455	Other		x						
oth19	NOVEL/CGI-49 PROTEIN	4929567	Other	x	x	x		x	x	x	x
oth20	NOVEL/CGI-94 PROTEIN	18539291	Other	x	x	x	x	x	x	x	x
oth21	NOVEL/KIAA0102	7661908	Other	x	x		x	x			
oth22	NOVEL/KIAA0377	7662084	Other	x	x	x	x	x	x	x	x
oth23	P120 CATENIN ISOFORM 4B	3152867	Other	x	x	x		x	x		x
oth24	PAR INTERACTIN ASSOCIATED G PROTEIN	7514041	Other	x	x	x		x			
oth25	TORSIN A	10798614	Other		x						
sec1	ACZONIN/PICCOLO HOMOLOG	15139362	Secretory & Membrane Associated								
sec2	ADP-RIBOSYLATION FACTOR-LIKE 1	4502227	Secretory & Membrane Associated	x	x	x	x	x	x		x
sec3	ANNEXIN V	999937	Secretory & Membrane Associated	x	x	x	x	x		x	x
sec4	ANNEXIN VI	113962	Secretory & Membrane Associated	x	x	x	x	x		x	x
sec5	ANNEXIN VII	4502111	Secretory & Membrane Associated	x	x	x	x	x		x	x
sec6	ATAXIN 2 RELATED PROTEIN	6005699	Secretory & Membrane Associated		x						
sec7	BIP	6470150	Secretory & Membrane Associated	x	x	x	x	x	x	x	x
sec8	CALCYCLIN	116509	Secretory & Membrane Associated								
sec9	CALM PROTEIN/CLATHRIN ASSEMBLY LYMPHOIDMYELOID LEUKEMIA GENE	6005733	Secretory & Membrane Associated	x	x	x	x	x			x
sec10	CALNEXIN	6671664	Secretory & Membrane Associated	x	x	x	x	x		x	x
sec11	CALPACTIN 1 / ANNEXIN II ASSOCIATED LIGHT CHAIN	6677833	Secretory & Membrane Associated								
sec12	CALRETICULIN	253851	Secretory & Membrane Associated	x	x	x	x	x		x	x
sec13	CLATHRIN COAT ASSEMBLY PROTEIN AP50	113332	Secretory & Membrane Associated	x	x	x	x	x	x	x	x
sec14	CLATHRIN HEAVY CHAIN	1705915	Secretory & Membrane Associated	x	x	x	x	x	x	x	x
sec15	CONTACTIN ASSOCIATED 1/SIDEKICK HOMOLOG	6680954	Secretory & Membrane Associated		x						
sec16	COPI COATOMER COMPLEX, ALPHA SUBUNIT	2494888	Secretory & Membrane Associated	x	x	x	x	x	x	x	x
sec17	COPI COATOMER COMPLEX, BETA SUBUNIT	8571380	Secretory & Membrane Associated	x	x	x		x		x	
sec18	COPINE I	10719953	Secretory & Membrane Associated	x	x		x	x	x	x	x
sec19	DYNAMIN 2	1706539	Secretory & Membrane Associated	x	x	x	x	x	x	x	x
sec20	DYNAMIN-LIKE I	16758468	Secretory & Membrane Associated	x	x	x	x	x	x	x	x
sec21	DYNAMIN-LIKE PROTEIN DLP1	4868358	Secretory & Membrane Associated	x	x	x	x	x	x	x	x
sec22	ECM29P-LIKE	2224677	Secretory & Membrane Associated	x	x	x	x	x			
sec23	EH-DOMAIN CONTAINING 1/EHD-1	7106303	Secretory & Membrane Associated	x	x		x	x	x	x	
sec24	ENDOPHILIN B1/SH3GLB2	9910352	Secretory & Membrane Associated		x						
sec25	ENDOPLASMIN PRECURSOR/GRP94	108003	Secretory & Membrane Associated	x	x	x	x	x	x		x
sec26	FLOTILLIN 1	6679809	Secretory & Membrane Associated		x						
sec27	GLUT1 TRANSPORTER C-TERMINAL BINDING PROTEIN	3808216	Secretory & Membrane Associated		x						
sec28	GLUT4 VESICLE PROTEIN	4200444	Secretory & Membrane Associated		x						
sec29	GMX33/GOLPH3	8922589	Secretory & Membrane Associated		x	x					
sec30	GOLGI STACKING PROTEIN HOMOLOG 55	5901572	Secretory & Membrane Associated	x	x	x	x		x	x	
sec31	GUANINE NUCLEOTIDE BINDING PROTEIN, ALPHA 14/G ALPHA 14	6680035	Secretory & Membrane Associated	x	x	x	x	x			
sec32	HUNTINGTIN-ASSOCIATED PROTEIN INTERACTIN ASSOCIATED GPROTEIN HIP	4504335	Secretory & Membrane Associated		x						
sec33	MYOFERLIN/FER-1 LIKE PROTEIN	10834587	Secretory & Membrane Associated		x						
sec34	MYOSIN VI	6678992	Secretory & Membrane Associated	x	x	x	x	x	x	x	x
sec35	NIPSNAP1	7512727	Secretory & Membrane Associated		x						
sec36	NSF	6679140	Secretory & Membrane Associated	x	x	x	x	x	x	x	x
sec37	PHOSPHOLIPASE C BETA 2	3688530	Secretory & Membrane Associated	x	x	x	x	x	x	x	x
sec38	RAB GDI ALPHA	1707886	Secretory & Membrane Associated	x	x	x	x	x	x	x	x
sec39	RAB22	9963781	Secretory & Membrane Associated	x	x	x	x	x	x	x	x
sec40	RAB6-KIFL	6225915	Secretory & Membrane Associated		x	x					
sec41	RAB7	4105819	Secretory & Membrane Associated	x	x	x	x	x	x	x	x
sec42	RACK 1	1083582	Secretory & Membrane Associated	x	x	x	x	x	x	x	x
sec43	SEC13	2498892	Secretory & Membrane Associated	x	x	x	x	x	x	x	x
sec44	SEC23	5454044	Secretory & Membrane Associated	x	x	x	x	x	x	x	x
sec45	SEC31	7715039	Secretory & Membrane Associated	x	x	x	x	x	x	x	x
sec46	SEC3P-LIKE	8922746	Secretory & Membrane Associated	x	x	x	x	x			
sec47	SORTING NEXIN 4	10720282	Secretory & Membrane Associated	x	x	x		x	x	x	x
sec48	STAUFEN	6755674	Secretory & Membrane Associated		x						
sec49	TMP21 PRECURSOR/ERV25P	7513284	Secretory & Membrane Associated	x	x	x	x	x	x	x	x
sec50	TORSIN B	10798612	Secretory & Membrane Associated		x						
sec51	VACULAR SORTING VPS35P	10435637	Secretory & Membrane Associated	x	x	x	x	x	x	x	x
sec52	VAMP-ASSOCIATED PROTEIN A	4240462	Secretory & Membrane Associated	x	x	x	x	x	x	x	
sec53	PATCHED	6679519	Secretory & Membrane Associated		x						
