# Supplementary material for: A Complex Cell Division Machinery Was Present in the Last Common Ancestor of Eukaryotes
Source: PLoS One. 2009 Apr 7;4(4):e5021. doi: 10.1371/journal.pone.0005021 (PMC2661371; doi:10.1371/journal.pone.0005021)
Supplement: Table S2 — Functional domain composition of the mammalian midbody proteins. This table shows the domain composition of each of the 155 components of the hamster midbody. The name and the PFAM accession number of each domain are provided. (0.06 MB PDF) [file pone.0005021.s008.pdf]

Supplementary Information Table S2. Functional domain composition of the mammalian midbody proteins.

Short name	Mammalian functionnal domains					
act1	Actin (PF00022.9)					
act2	Spectrin (PF00435.11)	SH3_1 (PF00018.18)	efhand (PF00036.22)	efhand_Ca_insen (PF08726.1)	SH3_2 (PF07653.7)	
act3	Actin (PF00022.9)					
act4	WD40 (PF00400.22)					
act5	Actin (PF00022.9)					
act6	G-patch (PF01585.13)	TFP11 (PF08697.1)				
act7	Cofilin_ADF (PF00241.10)					
act8	Myosin_head (PF00063.11)	IQ (PF00612.17)	Myosin_TH1 (PF06017.4)	SH3_1 (PF00018.18)		
act9	Myosin_N (PF02736.9)	Myosin_head (PF00063.11)	IQ (PF00612.17)	Myosin_tail_1 (PF01576.9)	Filament (PF00038.11)	
act10	WW (PF00397.16)	IQ (PF00612.17)	RasGAP (PF00616.9)	RasGAP_C (PF03836.5)		
act11	efhand (PF00036.22)					
act12	F_actin_cap_B (PF01115.8)					
act13	LIM (PF00412.12)					
act14	Filament_head (PF04732.4)	Filament (PF00038.11)				
act15	HEAT (PF02985.12)	Arm (PF00514.13)				
act16	CH (PF00307.21)	Spectrin (PF00435.11)	efhand (PF00036.22)	GAS2 (PF02187.8)	Tropomyosin (PF00261.10)	DUF1690 (PF07956.2)
act17	Band_41 (PF00373.8)	ERM (PF00769.9)				
act18	Cofilin_ADF (PF00241.10)					
act19	P21-Arc (PF04062.5)					
act20	Band_41 (PF00373.8)	ERM (PF00769.9)				
act21	Band_41 (PF00373.8)	Talin_middle (PF09141.1)	VBS (PF08913.1)	I_LWEQ (PF01608.8)		
act22	Septin (PF00735.9)					
act23	efhand (PF00036.22)	CH (PF00307.21)				
act24	BRCT (PF00533.16)	RhoGEF (PF00621.11)	PH (PF00169.19)			
act25	Band_41 (PF00373.8)	FA (PF08736.1)	SAB (PF04382.3)	4_1_CTD (PF05902.3)		
act26	CH (PF00307.21)	Filamin (PF00630.9)				
act27	CH (PF00307.21)	Spectrin (PF00435.11)	PH (PF00169.19)			
act28	CH (PF00307.21)	Spectrin (PF00435.11)	efhand (PF00036.22)	efhand_Ca_insen (PF08726.1)		
act29	P34-Arc (PF04045.5)					
act30	CH (PF00307.21)	Calponin (PF00402.8)				
act31	Drf_GBD (PF06371.4)	Drf_FH3 (PF06367.7)	FH2 (PF02181.13)			
act32	Ras (PF00071.12)	Arf (PF00025.11)				
act34	Band_41 (PF00373.8)	ERM (PF00769.9)				
act35	Vinculin (PF01044.9)	MCPsignal (PF00015.11)	ArgJ (PF01960.9)			
act36	DUF1899 (PF08953.1)	WD40 (PF00400.22)	DUF1900 (PF08954.1)			
act37	Septin (PF00735.9)	MMR_HSR1 (PF01926.13)				
act38	Profilin (PF00235.9)					
act39	TBPIP (PF07106.3)					
act40	Extensin_2 (PF04554.4)	CH (PF00307.21)				
act41	PDZ (PF00595.14)	SH3_2 (PF07653.7)				
act42	efhand (PF00036.22)					
act43	PDZ (PF00595.14)	LIM (PF00412.12)				
act44	Actin (PF00022.9)					
act45	BAR (PF03114.8)	RhoGAP (PF00620.17)				
act46	BTB (PF00651.21)	BACK (PF07707.5)	Kelch_1 (PF01344.15)	Kelch_2 (PF07646.5)		
kin1	Pkinase (PF00069.15)					
kin2	Pkinase (PF00069.15)					
kin3	CK_II_beta (PF01214.9)					
kin4	Pkinase (PF00069.15)					
kin5	CKS (PF01111.9)					
kin7	Pkinase (PF00069.15)	Pkinase_C (PF00433.14)	ATG16 (PF08614.1)	C1_1 (PF00130.12)	PH (PF00169.19)	CNH (PF00780.12)
kin8	*No domain detected above threshold*					
kin9	Pkinase (PF00069.15)	HEAT (PF02985.12)				
kin10	Nucleoplasmin (PF03066.5)					
kin11	Pkinase (PF00069.15)	POLO_box (PF00659.9)				
kin12	Metallophos (PF00149.18)					
kin13	HEAT (PF02985.12)					
kin14	Metallophos (PF00149.18)					
kin15	LRR_1 (PF00560.23)					
kin16	LRR_1 (PF00560.23)					

kin17	Pkinase (PF00069.15)					
kin18	Pkinase (PF00069.15)	UVR (PF02151.9)	Pkinase_Tyr (PF07714.7)			
mic1	Tubulin (PF00091.15)	Tubulin_C (PF03953.7)				
mic2	Tubulin (PF00091.15)	Tubulin_C (PF03953.7)				
mic3	Kinesin (PF00225.13)	DHC_N1 (PF08385.2)				
mic4	CAP_GLY (PF01302.15)	bZIP_1 (PF00170.11)	Myosin_tail_1 (PF01576.9)			
mic5	CAP_GLY (PF01302.15)					
mic6	Dynamitin (PF04912.4)	OKR_DC_1_C (PF03711.5)				
mic7	DHC_N1 (PF08385.2)	DHC_N2 (PF08393.3)	AAA_5 (PF07728.4)	Dynein_heavy (PF03028.6)		
mic8	CH (PF00307.21)	EB1 (PF08393.3)				
mic9	Spc97_Spc98 (PF04130.4)					
mic10	Kinesin (PF00225.13)	ERM (PF00769.9)				
mic11	Kinesin (PF00225.13)					
mic12	Kinesin (PF00225.13)					
mic13	Rab5-bind (PF09311.1)	TPR_2 (PF07719.7)	TPR_1 (PF00515.18)			
mic14	MAP1B_neuraxin (PF00414.8)					
mic15	Kinesin (PF00225.13)					
mic16	Kinesin (PF00225.13)					
mic17	HEAT (PF02985.12)					
mic18	HEAT (PF02985.12)					
oth1	WD40 (PF00400.22)					
oth2	Siah-Interact_N (PF09032.1)	CS (PF04969.6)	SGS (PF05002.5)			
oth3	S_100 (PF01023.9)					
oth4	TPR_1 (PF00515.18)					
oth5	WD40 (PF00400.22)					
oth6	CDI (PF02234.9)					
oth7	Cullin (PF00888.12)					
oth8	ER (PF01133.8)					
oth9	WD40 (PF00400.22)					
oth10	GDNF (PF02351.6)					
oth11	G-alpha (PF00503.10)					
oth12	Filament (PF00038.11)					
oth13	Filament (PF00038.11)					
oth14	Mago_nashi (PF02792.5)					
oth15	MAPKK1_Int (PF08923.1)					
oth16	C2 (PF00168.20)	WW (PF00397.16)	HECT (PF00632.15)			
oth17	Noggin (PF05806.3)					
oth18	WH1 (PF00568.13)	VASP_tetra (PF08776.1)				
oth19	Saccharop_dh (PF03435.8)					
oth20	Utp11 (PF03998.4)					
oth21	SPC25 (PF06703.2)					
oth22	Acid_phosphat_A (PF00328.12)					
oth23	Arm (PF00514.13)					
oth24	DNA_pol_phi (PF04931.4)					
oth25	Torsin (PF06309.2)					
sec1	zf-piccolo (PF05715.3)	Extensin_2 (PF04554.4)	PDZ (PF00595.14)	C2 (PF00168.20)		
sec2	Arf (PF00025.11)					
sec3	Annexin (PF00191.10)					
sec4	Annexin (PF00191.10)					
sec5	Annexin (PF00191.10)					
sec6	Ataxin-2_N (PF06741.3)	PAM2 (PF07145.5)				
sec7	HSP70 (PF00012.10)					
sec8	S_100 (PF01023.9)	efhand (PF00036.22)				
sec9	ANTH (PF07651.6)					
sec1012	Calreticulin (PF00262.8)					
sec11	S_100 (PF01023.9)					
sec13	Clat_adaptor_s (PF01217.10)	Adap_comp_sub (PF00928.11)				
sec14	Clathrin_propel (PF01394.10)	Clathrin (PF09268.1)	Clathrin (PF00637.10)			
sec15	ig (PF00047.15)	I (PF07679.6)	fn3 (PF00041.11)	V-set (PF07686.7)		
sec16	WD40 (PF00400.22)	Coatomer_WDAD (PF04053.4)	COPI_C (PF06957.2)			
sec17	Adaptin_N (PF01602.10)	Coatamer_beta_C (PF07718.3)				
sec18	C2 (PF00168.20)	Copine (PF07002.6)				

sec192021	Dynamin_N (PF00350.13)	Dynamin_M (PF01031.10)	GED (PF02212.8)			
sec22	HEAT (PF02985.12)					
sec23	Dynamin_N (PF00350.13)	efhand (PF00036.22)				
sec24	BAR (PF03114.8)	SH3_1 (PF00018.18)	IMD (PF08397.1)	SH3_2 (PF07653.7)		
sec25	HATPase_c (PF02518.16)	HSP90 (PF00183.9)				
sec26	Band_7 (PF01145.15)	Flotillin (PF03149.6)				
sec27	PDZ (PF00595.14)					
sec28	C2 (PF00168.20)					
sec29	GPP34 (PF05719.2)					
sec30	GRASP55_65 (PF04495.4)					
sec31	G-alpha (PF00503.10)	Arf (PF00025.11)				
sec32	Spectrin (PF00435.11)	RhoGEF (PF00621.11)	PH (PF00169.19)			
sec33	C2 (PF00168.20)	FerI (PF08151.2)	FerA (PF08165.2)	FerB (PF08150.2)		
sec34	Myosin_head (PF00063.11)	IQ (PF00612.17)				
sec35	NIPSNAP (PF07978.3)					
sec36	CDC48_N (PF02359.8)	CDC48_2 (PF02933.7)	AAA (PF00004.19)			
sec37	efhand_like (PF09279.1)	PI (PF00388.9)	PI-PLC-Y (PF00387.9)	C2 (PF00168.20)	PLC-beta_C (PF08703.1)	
sec38	GDI (PF00996.9)					
sec39	Ras (PF00071.12)					
sec40	Kinesin (PF00225.13)					
sec41	Ras (PF00071.12)					
sec42	WD40 (PF00400.22)					
sec43	WD40 (PF00400.22)					
sec44	zf-Sec23_Sec24 (PF04810.5)	Sec23_trunk (PF04811.5)	Sec23_BS (PF08033.2)	Sec23_helical (PF04815.5)	Gelsolin (PF00626.12)	
sec45	WD40 (PF00400.22)	Extensin_2 (PF04554.4)				
sec46	Vps52 (PF04129.3)					
sec47	PX (PF00787.14)	Vps5 (PF09325.1)				
sec48	dsrm (PF00035.15)					
sec49	*No domain detected above threshold*					
sec50	Torsin (PF06309.2)					
sec51	Vps35 (PF03635.7)					
sec52	Motile_Sperm (PF00635.16)					
sec53	Patched (PF02460.9)					
