# Supplementary material for: A Complex Cell Division Machinery Was Present in the Last Common Ancestor of Eukaryotes
Source: PLoS One. 2009 Apr 7;4(4):e5021. doi: 10.1371/journal.pone.0005021 (PMC2661371; doi:10.1371/journal.pone.0005021)
Supplement: Table S3 — Comparison of the functional domain composition of mammalian midbody proteins with their non metazoan eukaryotic orthologues. Comparison with the domain composition of the orthologues found in Aspergillus fumigatus, Neurospora crassa, Cryptococcus neoformans, Ustilago maydis, Saccharomyces cerevisiae, Schizosaccharomyces pombe, Dictyostelium. Discoideum, Entamoeba histolytica, Arabidopsis thaliana, Oryza sativa, Ostreococcus tauri, Paramecium tetraurelia, Cryptosporidium hominis, Plasmodium falciparum, Theileria annulata, Phytophtora Ramorum, Thalassiosira pseudonana, Trypanosoma brucei, Leishmania major, Giardia lamblia and Trichomonas vaginalis. Asterisks indicate orthologues having the same domain composition as their mammalian counterparts; dollars designate orthologues displaying at least one difference with their mammalian counterparts; whereas "no" indicate that no orthologues are available. (0.27 MB PDF) [file pone.0005021.s009.pdf]

Supplementary Information Table S3.
Comparison with the domain composition of the orthologues found in various eukaryotes

Short name	*S. cerevisiae*	*S. pombe*	*Cryptococcus neoformans*	*Ustilago maydis*	*Dictyostelium discoideum*	*Entamoeba histolytica*	*A. thaliana*	*O.sativa*	*Ostreococcus tauri*	*Paramecium tetraurelia*	*Cryptosporidium hominis*	*Plasmodium falciparum*	*Theileria annulata*	*Phytophtora ramorum*	*Thalassiosira pseudonana*	*Trypanosoma brucei*	*Leishmania major*	*Trichomonas vaginalis*	*Giardia lamblia*	*Aspergillus fumigatus*	*Neurospora crassa*
act1	*	*	*	*	*	*	*	*	*	*	*	*	*	*	*	*	*	*	*	*	*
act2	no	no	no	no	no	no	no	no	no	no	no	no	no	no	no	no	no	no	no	no	no
act3	*	*	*	*	*	*	*	*	*	*	no	no	no	*	no	*	*	*	no	*	*
act4	*	*	*	*	*	*	*	*	no	*	*	no	no	*	no	*	no	*	no	*	*
act5	*	*	*	*	*	*	*	*	*	*	no	no	no	*	no	*	*	*	no	*	*
act6	no	*	*	*	*	no	*	*	*	no	no	no	no	*	$	no	no	no	no	*	no
act7	*	*	*	*	*	*	*	*	*	*	*	*	*	*	*	*	*	*	no	*	*
act8	$	$	$	$	$	$	$	$	$	$	$	$	$	no	$	$	$	no	no	$	$
act9	$	$	$	$	$	$	no	no	no	no	no	no	no	no	no	no	no	no	no	$	$
act10	*	*	$	$	$	$	no	no	no	no	no	no	no	no	no	no	no	no	no	$	$
act11	*	no	*	*	*	*	no	no	no	no	no	no	no	no	no	no	no	no	no	*	*
act12	no	*	*	*	*	*	*	*	no	*	no	*	no	*	no	no	no	*	no	*	*
act13	no	no	no	no	no	no	no	no	no	no	no	no	no	no	no	no	no	no	no	no	no
act14	no	no	no	no	no	no	no	no	no	no	no	no	no	no	no	no	no	no	no	no	no
act15	no	no	no	no	no	no	no	no	no	no	no	no	no	no	no	no	no	no	no	no	no
act16	no	no	no	no	no	no	no	no	no	no	no	no	no	no	no	no	no	no	no	no	no
act17	no	no	no	no	no	no	no	no	no	no	no	no	no	no	no	no	no	no	no	no	no
act18	no	$	$	$	no	$	no	no	no	no	no	no	no	no	no	no	no	no	no	$	$
act19	*	*	*	*	*	*	*	*	no	*	no	no	no	*	no	*	no	*	no	*	*
act20	no	no	no	no	no	no	no	no	no	no	no	no	no	no	no	no	no	no	no	no	no
act21	no	no	no	no	no	no	no	no	no	no	no	no	no	no	no	no	no	no	no	no	no
act22	*	$	*	*	no	no	no	no	no	no	no	no	no	no	no	no	no	no	no	*	*
act23	$	*	$	*	*	no	$	$	no	$	no	no	no	$	no	no	no	*	no	$	$
act24	no	no	no	no	no	no	no	no	no	no	no	no	no	no	no	no	no	no	no	no	no
act25	no	no	no	no	no	no	no	no	no	no	no	no	no	no	no	no	no	no	no	no	no
act26	no	no	no	no	no	no	no	no	no	no	no	no	no	no	no	no	no	no	no	no	no
act27	no	no	no	no	no	no	no	no	no	no	no	no	no	no	no	no	no	no	no	no	no
act28	no	*	*	$	$	$	no	no	no	no	no	no	no	$	no	no	no	$	no	$	$
act29	*	*	*	*	*	*	*	*	*	*	no	no	no	$	no	*	no	*	no	*	*
act30	no	no	no	no	no	no	no	no	no	no	no	no	no	no	no	no	no	no	no	no	no
act31	*	$	$	$	$	no	no	no	no	no	no	no	no	no	no	no	no	no	no	$	$
act32	$	*	*	*	no	no	no	no	no	no	no	no	no	no	no	no	no	no	no	$	*
act34	no	no	no	no	no	no	no	no	no	no	no	no	no	no	no	no	no	no	no	no	no
act35	no	no	no	no	no	no	no	no	no	no	no	no	no	no	no	no	no	no	no	no	no
act36	*	*	$	*	*	*	no	no	no	*	*	$	$	*	*	*	*	*	no	*	*
act37	*	*	$	*	no	no	no	no	no	*	no	no	no	no	no	no	no	no	no	*	*
act38	*	*	*	*	*	*	*	*	no	*	no	no	no	no	no	*	*	*	no	*	*
act39	*	*	*	no	no	no	*	*	*	*	no	no	no	*	no	*	no	*	*	no	no

act40	no	no	no	no	no	no	no	no	no	no	no	no	no	no	no	no	no	no	no	no	no
act41	no	no	no	no	no	no	no	no	no	no	no	no	no	no	no	no	no	no	no	no	no
act42	*	*	*	*	*	*	no	no	no	no	no	no	no	no	no	no	no	no	no	no	no
act43	no	no	no	no	no	no	no	no	no	no	no	no	no	no	no	no	no	no	no	no	no
act44	*	*	*	no	*	no	*	*	*	no	*	no	no	no	no	no	no	no	no	*	no
act45	no	no	no	no	no	no	no	no	no	no	no	no	no	no	no	no	no	no	no	no	no
act46	no	no	no	no	no	no	no	no	no	no	no	no	no	no	no	no	no	no	no	no	no
kin1	*	$	*	*	*	no	*	*	*	*	no	no	no	*	*	no	no	*	no	*	$
kin2	*	*	*	*	*	*	$	$	*	no	*	*	$	*	*	*	*	*	*	*	*
kin3	*	*	*	*	*	*	*	*	*	*	*	*	*	*	*	*	*	*	*	*	*
kin4	*	$	*	no	*	*	*	*	*	*	no	*	*	*	no	no	no	no	no	*	*
kin5	no	*	*	*	$	*	*	*	*	*	*	*	*	*	*	*	*	*	*	*	*
kin7	no	no	no	no	no	no	no	no	no	no	no	no	no	no	no	no	no	no	no	no	no
kin8	no	no	no	no	no	no	no	no	no	no	*	no	no	no	no	no	no	no	no	no	no
kin9	$	$	$	$	*	$	*	*	$	no	$	$	$	no	no	no	no	no	no	$	$
kin10	no	no	no	no	no	no	no	no	no	no	no	no	no	no	no	no	no	no	no	no	no
kin11	*	*	*	*	*	*	no	no	*	*	no	no	no	*	$	*	*	*	$	$	*
kin12	*	*	*	no	*	no	*	*	no	*	*	*	*	no	no	no	no	no	*	*	*
kin13	*	*	*	*	*	*	*	*	*	no	no	no	no	no	no	no	no	no	no	$	$
kin14	*	*	*	no	*	*	*	*	*	*	no	*	no	*	no	*	*	*	*	*	*
kin15	no	no	no	no	$	*	*	*	no	no	no	no	no	no	no	$	$	$	no	no	no
kin16	no	no	no	no	$	*	*	*	$	no	$	$	no	no	no	no	$	no	no	no	no
kin17	*	*	*	$	$	no	*	*	$	no	no	no	no	no	no	no	no	*	no	*	*
kin18	no	no	no	no	no	no	no	no	no	no	no	no	no	no	no	no	no	no	no	no	no
mic1	*	*	*	*	*	*	*	*	*	*	*	*	*	*	*	*	*	*	*	*	*
mic2	*	*	*	*	*	*	*	*	*	*	*	*	*	*	*	*	*	*	*	*	*
mic3	$	$	$	$	*	$	$	$	*	*	$	$	no	*	$	no	no	$	*	$	$
mic4	no	no	no	no	no	no	no	no	no	no	no	no	no	no	no	no	no	no	no	no	no
mic5	no	no	*	$	no	no	no	no	no	no	no	no	no	no	no	no	no	no	no	$	$
mic6	no	no	$	no	$	no	no	no	no	no	no	no	no	no	no	no	no	no	no	*	*
mic7	$	$	$	$	*	no	no	no	no	$	no	$	$	$	no	$	$	*	no	$	$
mic8	*	*	*	*	*	no	*	*	*	*	*	*	*	$	$	no	no	*	*	$	*
mic9	*	*	*	*	*	no	*	*	*	*	no	no	no	*	*	*	*	*	*	*	*
mic10	$	$	$	$	no	no	$	$	*	*	no	no	no	no	no	*	$	no	$	$	$
mic11	*	*	*	*	*	*	*	*	*	*	no	no	no	no	no	*	$	no	*	*	*
mic12	no	*	$	$	*	no	*	*	*	*	*	*	no	*	$	*	$	*	*	$	$
mic13	no	no	no	no	no	no	no	no	no	no	no	no	no	no	no	no	no	no	no	no	no
mic14	no	no	no	no	no	no	no	no	no	no	no	no	no	no	no	no	no	no	no	no	no
mic15	no	no	no	no	*	no	*	*	*	*	*	*	*	*	*	*	*	*	$	no	no
mic16	no	no	no	no	no	no	no	no	no	no	no	no	no	no	no	no	no	no	no	no	no
mic17	no	no	no	no	no	no	no	no	no	no	no	no	no	no	no	no	no	no	no	no	no
mic18	no	no	*	$	*	no	*	*	$	$	no	no	no	no	no	no	no	no	no	no	no
oth1	*	*	*	*	*	$	*	*	*	$	*	no	*	*	*	*	*	*	$	*	*
oth2	no	no	no	no	no	$	*	*	no	*	$	*	$	$	$	no	no	$	no	no	no
oth3	no	no	no	no	no	no	no	no	no	no	no	no	no	no	no	no	no	no	no	no	no
oth4	*	*	*	*	$	no	$	*	*	*	*	no	no	no	no	*	*	*	no	*	$
oth5	*	*	*	*	*	*	*	*	$	*	no	*	no	*	*	*	*	*	$	*	*
oth6	no	no	no	no	no	no	no	no	no	no	no	no	no	no	no	no	no	no	no	no	no
oth7	no	*	*	no	*	*	*	*	*	*	*	*	*	*	*	$	*	*	no	*	*

oth8	no	no	no	no	*	no	*	*	no	no	*	*	no	*	no	no	no	no	no	no	no
oth9	*	*	*	*	*	*	*	*	*	$	*	1	*	*	*	*	$	*	$	*	*
oth10	no	no	no	no	no	no	no	no	no	no	no	no	no	no	no	no	no	no	no	no	no
oth11	*	*	*	*	*	*	$	$	no	no	no	no	no	*	*	no	no	*	no	*	*
oth12	no	no	no	no	no	no	no	no	no	no	no	no	no	no	no	no	no	no	no	no	no
oth13	no	no	no	no	no	no	no	no	no	no	no	no	no	no	no	no	no	no	no	no	no
oth14	no	*	*	*	*	*	*	*	*	*	no	*	*	*	*	*	*	no	no	*	*
oth15	no	no	no	no	*	no	no	no	no	no	no	no	no	$	no	no	no	*	no	no	no
oth16	*	*	*	*	no	no	no	no	no	no	no	no	no	no	no	no	no	no	no	*	*
oth17	no	no	no	no	no	no	no	no	no	no	no	no	no	no	no	no	no	no	no	no	no
oth18	no	no	no	no	no	no	no	no	no	no	no	no	no	no	no	no	no	no	no	no	no
oth19	no	no	*	*	no	no	*	*	*	no	*	*	no	*	no	no	*	no	no	*	*
oth20	*	*	*	*	*	*	*	*	*	*	*	*	*	*	no	no	no	$	*	*	*
oth21	no	no	no	no	*	no	*	*	no	no	no	no	no	no	no	no	no	no	no	no	no
oth22	no	*	$	$	$	no	*	*	$	$	*	$	*	*	$	no	no	no	$	$	$
oth23	$	*	$	no	no	no	$	$	$	$	no	$	no	no	no	$	$	$	$	$	no
oth24	no	*	*	*	no	no	$	$	$	no	no	no	no	no	no	no	no	no	no	no	no
oth25	no	no	no	no	no	no	no	no	no	no	no	no	no	no	no	no	no	no	no	no	no
sec1	no	no	no	no	no	no	no	no	no	no	no	no	no	no	no	no	no	no	no	no	no
sec2	*	*	*	*	*	*	*	*	*	*	*	*	*	no	no	*	*	no	*	*	*
sec3	no	no	*	*	*	no	*	*	*	no	no	no	no	*	*	no	no	no	*	*	*
sec4	no	no	*	*	*	no	*	*	*	no	no	no	no	*	*	no	no	no	*	*	*
sec5	no	no	*	*	*	no	*	*	no	no	no	no	no	*	*	no	no	no	*	*	*
sec6	no	no	no	no	no	no	no	no	no	no	no	no	no	no	no	no	no	no	no	no	no
sec7	no	no	*	*	*	*	*	*	no	*	*	*	*	*	no	*	*	*	*	*	no
sec8	no	no	no	no	no	no	no	no	no	no	no	no	no	no	no	no	no	no	no	no	no
sec9	no	*	$	$	*	no	*	*	*	no	no	no	no	no	no	*	$	no	no	*	*
sec1012	no	*	*	no	*	*	*	*	$	no	no	no	no	*	no	no	no	*	no	*	*
sec11	no	no	no	no	no	no	no	no	no	no	no	no	no	no	no	no	no	no	no	no	no
sec13	*	*	*	*	*	$	$	$	$	$	no	no	no	$	$	no	no	$	*	*	*
sec14	*	*	*	*	*	*	*	*	$	$	$	$	$	*	$	*	$	$	$	$	*
sec15	no	no	no	no	no	no	no	no	no	no	no	no	no	no	no	no	no	no	no	no	no
sec16	*	*	*	*	*	$	*	*	*	*	*	*	*	$	*	*	*	*	*	*	*
sec17	$	$	*	$	no	no	$	$	$	no	no	no	no	$	$	no	no	no	no	*	*
sec18	no	no	no	no	*	no	*	*	no	*	no	no	no	*	no	no	$	*	no	no	no
sec192021	*	*	*	*	*	*	*	*	$	*	$	*	*	*	$	*	*	*	*	*	*
sec22	*	$	no	no	$	no	*	*	$	no	no	no	no	no	no	no	no	no	no	*	*
sec23	no	no	no	no	*	$	*	*	no	no	no	$	no	*	$	no	no	no	no	no	no
sec24	no	no	no	no	no	no	no	no	no	no	no	no	no	no	no	no	no	no	no	no	no
sec25	*	*	*	*	*	*	*	*	*	*	*	*	no	no	no	*	*	*	*	*	*
sec26	no	no	no	no	no	no	no	no	no	no	no	no	no	no	no	no	no	no	no	no	no
sec27	no	no	no	no	no	no	no	no	no	no	no	no	no	no	no	no	no	no	no	no	no
sec28	no	no	no	no	no	no	no	no	no	no	no	no	no	no	no	no	no	no	no	no	no
sec29	*	*	*	*	no	no	no	no	no	no	no	no	no	no	no	no	no	no	no	*	*
sec30	*	*	*	*	*	no	no	no	no	*	*	*	*	*	*	no	no	no	no	*	*
sec31	*	*	*	no	*	*	$	*	no	no	no	no	no	no	no	no	no	no	no	*	*
sec32	no	no	no	no	no	no	no	no	no	no	no	no	no	no	no	no	no	no	no	no	no
sec33	no	no	no	no	no	no	no	no	no	no	no	no	no	no	no	no	no	no	no	no	no
sec34	*	*	$	$	$	$	*	*	$	*	$	$	$	*	$	$	$	no	no	$	$

sec35	no	no	no	no	no	no	no	no	no	no	no	no	no	no	no	no	no	no	no	no	no
sec36	$	$	$	no	$	$	$	$	$	$	$	*	$	$	$	$	$	*	$	$	$
sec37	$	$	$	$	$	no	$	$	$	$	$	$	$	no	$	$	$	no	no	$	$
sec38	*	*	no	*	*	*	*	*	*	*	*	*	*	*	*	*	*	*	*	*	*
sec39	*	$	$	no	$	*	*	*	$	no	*	*	$	no	*	$	$	*	*	*	*
sec40	no	*	*	*	no	no	no	no	no	no	no	no	no	no	no	no	no	no	no	no	no
sec41	$	$	$	$	$	$	$	$	*	$	no	*	$	$	$	$	$	*	$	$	$
sec42	no	*	*	$	*	*	*	*	*	*	*	*	*	$	*	*	*	*	no	*	*
sec43	*	*	*	*	*	*	*	*	*	*	*	*	no	*	*	*	*	no	no	*	*
sec44	*	*	*	*	*	*	*	*	*	*	*	*	*	*	*	*	*	*	$	*	*
sec45	$	$	*	*	*	*	$	$	$	*	$	*	*	$	no	*	*	*	no	*	*
sec46	no	no	$	$	$	no	$	$	no	no	no	no	no	no	no	no	no	no	no	$	$
sec47	*	no	*	no	no	no	*	*	no	$	no	no	no	*	no	*	no	no	no	*	$
sec48	no	no	no	no	no	no	no	no	no	no	no	no	no	no	no	no	no	no	no	no	no
sec49	*	*	*	*	$	*	$	$	*	*	*	*	*	$	no	*	$	no	$	*	*
sec50	no	no	no	no	no	no	no	no	no	no	no	no	no	no	no	no	no	no	no	no	no
sec51	*	no	*	*	*	*	*	*	*	*	*	*	*	*	*	*	*	*	*	*	*
sec52	*	*	*	*	*	no	*	*	*	*	no	no	no	*	no	no	no	no	no	*	*
sec53	no	no	no	no	no	no	no	no	no	no	no	no	no	no	no	no	no	no	no	no	no
