# Supplementary material for: A Complex Cell Division Machinery Was Present in the Last Common Ancestor of Eukaryotes
Source: PLoS One. 2009 Apr 7;4(4):e5021. doi: 10.1371/journal.pone.0005021 (PMC2661371; doi:10.1371/journal.pone.0005021)
Supplement: Table S5 — Components of the midbody, fungal septum division and phragmoplast studied by Otegui et al. and their relations of orthology. Grey columns correspond to proteins studied by Otegui et al. in mammals, Saccharomyces cerevisiae, Schizosaccharomyces pombe and plants [17]. Orthologues of these components present in the hamster midbody proteome are indicated by a cross. Dollars indicate paralogues of components of the hamster midbody. (0.04 MB PDF) [file pone.0005021.s011.pdf]

Supplementary Information Table S5. Components of the midbody, fungal septum division and phragmoplast studied by Otegui et *al.* and their relations of orthology.

Mammals		*S.pombe*		*S.cerevisiae*		Plants	
**From Otegui 2005**	**Present in the mammalian midbody proteome**	**From Otegui 2005**	**Orthologues of mammalian midbody components**	**From Otegui 2005**	**Orthologues of mammalian midbody components**	**From Otegui 2005**	**Orthologues of mammalian midbody components**
**AuroraB/AIM-1**	Aurora kinase B (kin1)	**Ark1p**	x	**Ipl1p**	x	**At-Aurora 1, -2**	x
**EB1**	EB1 (mic8)	**Mal3p**	x	**Bim1p**	x	**AtEB1 (A, B, C)**	x
**KIFC1/CHO2,<br>KIFC2,KIFC3**	KIFC1/KAR3P HOMOLOG (mic11)<br>paralogues of KIFC1/KAR3P (mic11)	**Pkl1p<br>Klp2p**	x<br>x	**Kar3p**	x	**AtK1/AtKatA, AtK2/AtKatB,<br>AtK3/AtKAtC, AtK4/AtKatD,<br>AtK5, AtKCBP**	no, x<br>x, x<br>x, no
**BimC**	BIMC KINESIN/EG5 (mic3)	**Cut7p**	x	**Kip1p<br>Cin8p**	x<br>$	**TKRP125,<br>DcKRP120–2**	x<br>x
**PLK1–4**	POLO-LIKE KINASE (kin11)	**Plo1p**	x	**Cdc5p**	x	**At4g24400**	$
**Dynamin II**	Dynamin (sec192021)	**Dnm1p**	x	**Dnm1p**	x	**DRP2Ab,<br>AtDRP1/Phragmoplastin,<br>ADL1A,<br>ADL1E**	$<br>$<br>$<br>$
**ch-TOG/XMAP215 homolog**	TOG /XMAP215 HOMOLOG (mic18)	**Dis1p<br>Alp14p**	$<br>$	**Stu2p**	$	**MOR1/GEM1**	x
**KIF4**	KIF4 (mic10)	**–**		**–**		**FRA1/At5g47820,<br>At3g50170,<br>At5g60930**	x<br>x<br>x
**CLIP-170/Restin**	CLIP-170 (mic4)	**Tip1p**		**Bik1p**		**MCLIP-170**	
**MKLP1/CHO1,<br>RabK6/MKLP2**	MKLP1 (mic16)	**–**		**–**		**At1g20060 (AtT20H2.17)**	
**p34cdc2/CDK1**	paralogue of CDC2 KINASE (kin4)	**Cdc2p**	$	**Cdc28p**	$	**CDC2**	$
**Formin/mDia**		**Bni1p**		**Cdc12p**		**AtFH5**	$
**TD-60**		**Pim1p**		**–**		**At1g19880**	
**Syntaxin 2**		**–**		**–**		**KNOLLE/AtSYP111**	
**Borealin/CDCa8**		**–**		**–**		**–**	
**TACC1**		**–**		**–**		**–**	
**Cdc14a**		**Cdc14p,<br>Clp1p/Flp1p**		**Cdc14p**		**–**	
**PRC1**		**Ase1p**		**Ase1p**		**MAP-65**	
**MgcRacGAP**		**Rga7p**		**Rgd1p**		**At4g24580**	
**INCENP**		**Pic1p**		**Sli15p**		**–**	
**Survivin**		**Bir1p/Cut17p**		**Bir1p**		**–**	
